# Single-site catalyst promoters accelerate metal-catalyzed nitroarene hydrogenation

**DOI:** 10.1038/s41467-018-03810-y

**Published:** 2018-04-10

**Authors:** Liang Wang, Erjia Guan, Jian Zhang, Junhao Yang, Yihan Zhu, Yu Han, Ming Yang, Cheng Cen, Gang Fu, Bruce C. Gates, Feng-Shou Xiao

**Affiliations:** 1 Key Laboratory of Applied Chemistry of Zhejiang Province, Department of Chemistry, Zhejiang University, Hangzhou, 310028 China; 2 Department of Materials Science and Engineering, University of California, Davis, CA 95616 United States; 3 State Key Laboratory for Catalysis, Dalian Institute of Chemical Physics, Chinese Academy of Science, Dalian, 116023 China; 4 Advanced Membranes and Porous Materials Center, Physical Sciences and Engineering Division, King Abdullah University of Science and Technology, Thuwal, 23955-6900 Saudi Arabia; 5 Department of Physics and Astronomy, West Virginia University, Morgantown, WV 26506-6315 United States; 6 State Key Laboratory for Physical Chemistry of Solid Surfaces, and National Engineering Laboratory for Green Chemical Productions of Alcohols-Ethers-Esters, College of Chemistry and Chemical Engineering, Xiamen University, Xiamen, 361005 China; 7 Department of Chemical Engineering, University of California, Davis, CA 95616 United States

## Abstract

Atomically dispersed supported metal catalysts are drawing wide attention because of the opportunities they offer for new catalytic properties combined with efficient use of the metals. We extend this class of materials to catalysts that incorporate atomically dispersed metal atoms as promoters. The catalysts are used for the challenging nitroarene hydrogenation and found to have both high activity and selectivity. The promoters are single-site Sn on TiO_2_ supports that incorporate metal nanoparticle catalysts. Represented as M/Sn-TiO_2_ (M = Au, Ru, Pt, Ni), these catalysts decidedly outperform the unpromoted supported metals, even for hydrogenation of nitroarenes substituted with various reducible groups. The high activity and selectivity of these catalysts result from the creation of oxygen vacancies on the TiO_2_ surface by single-site Sn, which leads to efficient, selective activation of the nitro group coupled with a reaction involving hydrogen atoms activated on metal nanoparticles.

## Abstract (web summary)

Understanding of the structures and roles of catalyst promoters markedly lags behind the understanding of the structures and roles of catalytic sites. Here, the authors address this challenge by incorporating a single-site promoter—tin—on a TiO_2_ surface to enhance the catalytic activity of various metals on the TiO_2_ in selective hydrogenation of nitroarenes.

## Introduction

Metal catalysts dispersed on solid supports dominate the technology of production of chemicals, fuels, and polymers, and they are the keys to environmental protection by clean-up of effluent gases from motor vehicles and fossil fuel power plants^1–4^. Hydrogenations are among the important technological reaction catalyzed by supported metals^5–10^. An important example is the hydrogenation of substituted nitroarenes, a widely used route for producing functionalized anilines as intermediates for the production of agrochemicals and antidetonators and as building blocks for high-performance rubbers and polymers^11,12^. In nitroarene hydrogenation, it is difficult to control the selectivity when more than one reducible group is present in the reactant^10,13–24^. The hydrogenation usually occurs preferentially on the non-target groups, giving low yields of the desired functionalized anilines. Important progress toward improved selectivity has been achieved by using gold-, cobalt oxide-, and ferric oxide-containing catalysts^1,10,13–15^, but these have the drawback of low activities, requiring high temperatures and long reaction times. Alternatively, the platinum-group metals offer the advantage of high catalytic activity, but their selectivities are generally low.

The performance of many prototypical supported metal catalysts (e.g., for industrial ammonia synthesis^25,26^, Fischer–Tropsch synthesis^27,28^, and the water-gas shift^29^) is enhanced markedly by promoters—additives that by themselves are not good catalysts. Understanding of the structures and roles of promoters markedly lags the understanding of catalysts. Important scientific foundations for understanding the roles of promoters have emerged from ultrahigh vacuum surface-science experiments with single metal crystals, but the results fall short of providing general guidance for the control of surface structures of supported catalysts.

We addressed this challenge in the context of selective hydrogenation of substituted nitroarenes and now report a general and efficient strategy to enhance the catalytic activity and/or selectivity of various metals (e.g., Au, Ru, Pt, and Ni) supported on TiO_2_—having achieved this goal by incorporating a single-site promoter—tin—on the TiO_2_ surface by a wet-chemical method giving what we refer to as metal/Sn–TiO_2_ catalysts. Our investigation illustrates that the Sn–O–Ti linkage facilitates the formation of oxygen vacancies on TiO_2_, which easily convert nitro groups into nitroso groups. Our promotion strategy gives a family of catalysts providing enhanced performance. For example, gold nanoparticles, which are generally selective but poorly active catalysts, as well as ruthenium, platinum, and nickel nanoparticles, which are active but poorly selective, are all transformed by the single-site tin promotion into catalysts that are both active and selective. Multi-technique characterization data show that the single-site tin species are associated with oxygen vacancies on titania formed in a hydrogen atmosphere that facilitate selective adsorption and activation of nitro groups, leading to the high catalytic activity and selectivity.

## Results

### Catalyst synthesis and characterization

The tin sites were anchored to TiO_2_ (anatase) by grafting of dimethyl tin dichloride, followed by calcination to remove the methyl groups. Then gold nanoparticles were loaded onto the Sn-grafted anatase by a urea-assisted impregnation. The resultant catalyst, denoted Au/Sn–TiO_2_-123, had an Au loading of 0.7 wt% and a Ti/Sn atomic ratio of 123 (Supplementary Table 2).

The weak tin signals in energy dispersive X-ray spectra of Sn–TiO_2_-123 characterizing randomly selected nanoscale regions (Supplementary Fig. 1) indicate a high dispersion of tin on TiO_2_. The tin atoms remain highly dispersed after gold loading—the lack of peaks characteristic of SnO_2_ or metallic gold in the XRD pattern of Au/Sn–TiO_2_-123 (Supplementary Fig. 2a) indicates that gold and tin species are both highly dispersed on the TiO_2_. The existence of Sn species on the surface of anatase was confirmed by X-ray photoelectron spectroscopy (Supplementary Fig. 2b). High-angle annular dark-field scanning transmission electron microscopy images of Au/Sn–TiO_2_-123 (Supplementary Fig. 3a and b) show gold nanoparticles with a mean diameter of 4.0 nm—clearly distinguished from the TiO_2_ support by the *Z*-contrast; tin species are not observable (Supplementary Fig. 3b and c), consistent with their being extremely small.

**Fig. 1:**
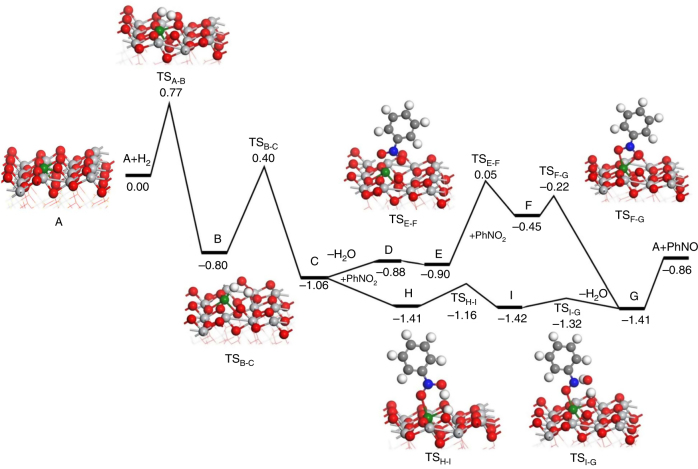
Energy profile of catalytic deoxygenation of nitrobenzene on Sn_1_/TiO_2_(101) surface. Color index: Ti, gray; Sn, green; O, red; C, dark gray; H, white; N, blue

The average coordination environments of tin in Sn–TiO_2_-123 and in Au/Sn–TiO_2_-123 were determined by extended X-ray absorption fine structure (EXAFS) spectroscopy, with data recorded at the Sn–K edge (Table 1, Supplementary Fig. 4). In contrast to the spectra of the references, tin foil and bulk SnO_2_ crystals (Supplementary Table 3), Sn–Sn contributions are absent from the spectra of both Sn–TiO_2_-123 and Au/Sn–TiO_2_-123, consistent with the presence of tin in isolated single sites. Correspondingly, the X-ray absorption near edge structure data indicate the presence of tin as cationic species (Supplementary Fig. 5). Two Sn–O shells, with coordination numbers of approximately 2 and 3, were found and denoted as Sn–O_1_ and Sn–O_2_, respectively (Table 1 and Fig. 1). The Sn–O_1_ and Sn–O_2_ contributions are assigned to Sn atoms bonded to oxygen atoms via different Sn–O–Ti linkages. Notably, the Sn–O_1_ bond length (2.0 Å) is less than that in bulk SnO_2_ crystals (2.05 Å, Supplementary Table 3), but the Sn–O_2_ bond length is greater (2.1 Å).

**Table 1:** EXAFS data characterizing Sn–TiO_2_-123 and Au/Sn–TiO_2_-123 under in situ treatment conditions

Sample	Shell	*N*	*R* (Å)	10^3^ × Δ*σ*^2^ (Å^2^)	Δ*E*_0_ (eV)
Sn–TiO_2_-123	Sn–O_1_	2.1	2.00	0.25	−7.99
	Sn–O_2_	2.9	2.10	2.35	5.22
	Sn–Ti_1_	1.1	3.08	2.88	8.46
	Sn–Ti_2_	1.5	3.50	7.00	0.07
Au/Sn–TiO_2_-123	Sn–O_1_	2.1	1.97	0.30	−6.75
	Sn–O_2_	3.0	2.07	0.40	9.42
	Sn–Ti_1_	1.0	3.08	2.25	12.7
	Sn–Ti_2_	2.3	3.49	8.38	2.18
H_2_ treated	Sn–O_1_	2.2	2.02	0.39	−3.93
Au/Sn–TiO_2_-123	Sn–O_2_	1.8	2.13	0.46	6.23
	Sn–Ti_1_	1.3	3.09	5.60	9.93
	Sn–Ti_2_	2.0	3.51	9.40	1.06
Nitrobenzene-adsorbed on Au/Sn–TiO_2_-123	Sn–O_1_	2.3	1.98	0.10	−7.99
	Sn–O_2_	3.0	2.09	1.32	7.90
	Sn–Ti_1_	1.0	3.07	5.05	6.94
	Sn–Ti_2_	2.4	3.49	13.0	6.40

EXAFS parameters characterizing Sn–TiO_2_-123 and Au/Sn–TiO_2_-123 (range of *k* = 3.44–12.08 Å^−1^, range of *R* = 0.5–4.0 Å); *N*, coordination number; *R*, distance between absorber and backscatterer atoms; Δ*σ*^2^, disorder term; Δ*E*_0_, inner potential correction. Error bounds (accuracies) characterizing the structural parameters obtained by EXAFS spectroscopy are estimated to be *N*, ± 15%; *R*, ± 0.02 Å; Δ*σ*^2^, ± 20%; Δ*E*_0_, ± 20%

Thus, to summarize, the EXAFS results provide evidence of single-site Sn in the Sn–TiO_2_-123 sample. Importantly, the single-site Sn is stable after loading of gold nanoparticles onto Sn–TiO_2_-123; the data show that single-site Sn in Au/Sn–TiO_2_-123 is in a coordination environment similar to that in Sn–TiO_2_-123 (Table 1).

### Catalyst performance

Our investigation of the catalytic properties of Au/Sn-TiO_2_-123 began with the hydrogenation of nitrobenzene as a prototype reaction (Table 2). The data show that the Sn–TiO_2_-123 sample without gold is inactive for the reaction, whereas Au/Sn–TiO_2_-123 gave complete nitrobenzene conversion in a short time of 1.5 h in a batch reactor at 373 K, with aniline as the sole product. In contrast, the Au/TiO_2_ sample without tin gave a nitrobenzene conversion of only 28.4% under the same conditions. Even after 10 h, the nitrobenzene conversion with Au/TiO_2_ was only 80.0%. Because these two catalysts had comparable gold loadings and nanoparticle sizes and the same TiO_2_ support (Supplementary Figs 3 and 6), we conclude that the enhanced catalytic activity of Au/Sn–TiO_2_-123 results from the presence of isolated Sn sites. Increasing the tin loading of Au/Sn–TiO_2_ to give a higher Sn/Ti ratio of 1/20 led to a significant decrease in the conversion of nitrobenzene, which is attributed to the formation of ineffective bulk SnO_2_ crystals, as shown by the XRD pattern (Supplementary Fig. 7).

**Table 2:** Catalytic data characterizing various Au catalysts in the hydrogenation of nitroarenes

	Catalyst	*T* (K)	Time (h)	Conversion (%)	Selectivity^a^ (%)	N balance closure^b^ (%)
Nitrobenzene reactant^c^						
	Sn–TiO_2_	373	1.5	—^d^	—	>99.5
	Au/Sn–TiO_2_-123	373	1.5	>99.5	>99.5	>99.5
	Au/TiO_2_	373	1.5	28.4	>99.5	>99.5
	Au/TiO_2_	373	10.0	80.0	>99.5	>99.5
	Au/Sn–TiO_2_-20	373	1.5	29.1	98.7	99.1
	Au/SnO_2_	373	10.0	52.0	99.1	99.0
3-Nitrostyrene reactant^e^						
	Au/TiO_2_	353	4.0	18.9	93.9	>99.5
	Au/TiO_2_	353	20.0	79.0	92.5	98.9
	Au/Sn–TiO_2_-123	353	4.0	99.0	99.3	>99.5
	Au/Sn–TiO_2_-20	353	20.0	24.2	91.2	>99.5
	Au/SnO_2_	353	20.0	18.0	75.1	>99.5
	Pt/TiO_2_	318	2.0	91.1	50.0	>99.5
	Pt/Sn–TiO_2_-123	318	2.0	98.5	97.4	>99.5
	Ru/TiO_2_	383	2.0	71.4	66.6	>99.5
	Ru/Sn–TiO_2_-123	383	2.0	99.0	98.4	98.5
	Ni/TiO_2_	393	4.5	5.7	51.7	98.5
	Ni/Sn–TiO_2_-123	393	4.5	52.0	90.1	99.2

^a^ Selectivity to the functionalized aniline

^b^ Calculated from the number of N-containing molecules in the reactor before and after the reaction

^c^ Reaction conditions: 0.5 mmol of nitroarene, 40 mg of catalyst, 4 mL of toluene, 1.3 MPa of H_2_

^d^ Undetectable

^e^ Reaction conditions: 0.5 mmol of nitroarene, 40 mg of catalyst, 4 mL of toluene, 0.2 MPa of H_2_ for Pt catalysts, 1.3 MPa of H_2_ for Ru catalysts and 2.5 MPa of H_2_ for Ni catalysts

When SiO_2_ was used instead of TiO_2_ as the support for gold nanoparticles, the resultant single-site Sn-modified supported gold catalyst (Au/Sn–SiO_2_-129, Supplementary Figs 8 and 9) was found to have a catalytic activity similar to that of the tin-free catalyst (Au/SiO_2_), demonstrating an essential role of TiO_2_ with the single-site Sn for achieving a highly active gold catalyst.

The selective hydrogenation of the nitro group in the presence of various other reducible substituent groups on the aromatic ring, including carbonyl, chloride, amide, vinyl, or nitrile, was investigated in the range 353–373 K by characterizing the hydrogenation of 3-nitrostyrene, 4-nitrobenzaldehyde, 4-nitrochlorobenzene, 2-chloro-4-nitrophenol, 4-nitrobenzamide, and 3-nitrobenzonitrile (Table 2 and Supplementary Table 4), respectively. The Au/TiO_2_ has been reported to be selective for the hydrogenation of 3-nitrostyrene^1^, with a selectivity of 93.9% to 3-vinylaniline at a conversion of 18.9% (3-ethylaniline was a byproduct). In contrast, 3-ethylaniline was barely detectable in the reaction catalyzed by Au/Sn–TiO_2_, giving a 3-vinylaniline selectivity of 99.3%. Significantly, the conversion of 3-nitrostyrene was much higher with Au/Sn–TiO_2_-123 (99.0%) than with Au/TiO_2_ (18.9%) under the same conditions, and the enhanced activity was further confirmed in our kinetics investigation (Supplementary Table 5). In the hydrogenation of other substituted nitroarenes, 2-chloro-4-nitrophenol, 4-nitrobenzaldehyde, 4-nitrochlorobenzene, 4-nitrobenzamide, and 3-nitrobenzonitrile, the Au/Sn–TiO_2_-123 catalyst always exhibited much higher activity than Au/TiO_2_. Even more significantly, the high selectivity to the corresponding substituted anilines was still maintained, or even further improved, with the promoted catalysts. These results demonstrate the excellent catalytic performance of Au/Sn–TiO_2_-123 and a decisive role of the site-isolated tin.

The efficient hydrogenation of nitroarenes promoted by single-site tin is not limited to gold catalysts; we extended the observations to platinum, ruthenium, and nickel catalysts, which are known to be active but poorly selective for the hydrogenation of substituted nitroarenes. For example, in the hydrogenation of 3-nitrostyrene (Table 2), TiO_2_-supported platinum nanoparticles (Pt/TiO_2_) exhibited a 3-nitrostyrene conversion of 91.1% with a 3-vinylaniline selectivity of 50.0%, and the Sn–TiO_2_-123-supported platinum nanoparticles (Pt/Sn–TiO_2_-123) gave a significantly increased 3-vinylaniline selectivity of 97.4% with a 3-nitrostyrene conversion of 98.5%. The kinetics data also demonstrate the enhancement of both activity and selectivity in the reaction catalyzed by Pt/Sn–TiO_2_-123 (Supplementary Table 5). In the hydrogenation of 2-chloro-4-nitrophenol (Supplementary Table 6), the Pt/Sn–TiO_2_-123 catalyst gave complete conversion of 2-chloro-4-nitrophenol with a 2-chloro-4-aminophenol selectivity of 99.0%. Under the same conditions, the Pt/TiO_2_ exhibited a much lower conversion (81.9%) and selectivity (56.0%) than the Pt/Sn–TiO_2_-123. Similar results were observed in the selective hydrogenation of 3-nitrostyrene (Tables 2) and 2-chloro-4-nitrophenol (Supplementary Table 6) with nickel and ruthenium catalysts, whereby the Ru/Sn–TiO_2_-123 and Ni/Sn-TiO_2_-123 always exhibited much higher selectivity than the conventional Ru/TiO_2_ and Ni/TiO_2_ catalysts. These results confirm the high efficiency and generality of the role of promotion by single-site tin on TiO_2_ in providing both highly active and highly selective TiO_2_-supported metal catalysts for the hydrogenation of substituted nitroarenes.

### Spectroscopy of working catalyst

To better understand the role of single-site Sn under reaction conditions, we did in-operando EXAFS spectroscopy characterizing H_2_- and nitrobenzene-treatments of the catalysts working at 373 K. For example, we found that the Sn–O coordination number representing the longer Sn–O bond in Au/Sn–TiO_2_-123 decreased significantly in the H_2_ treatment, from 3.0 to 1.8 (Table 1 and Supplementary Fig. 10), implying that the weak Sn–O linkages are partially cleaved in this treatment. When nitrobenzene was then introduced to the H_2_-treated Au/Sn–TiO_2_-123 sample, this coordination number increased to 3.0, leading us to assign the contribution to nitro groups interacting with Sn sites (Fig. 1). Notably, the average length of this Sn–O bond formed after introducing nitrobenzene was only 2.09 Å, confirming the efficient interaction.

Further experiments were carried out with Au/Sn–TiO_2_-123 by in situ Raman spectroscopy, which is widely used to investigate oxygen vacancies on TiO_2_^30,31^. As shown in Supplementary Fig. 11a, Au/Sn–TiO_2_-123 is characterized by the *E*_g_ mode of anatase at 143 cm^-1^, and this underwent a marked shift as a result of treatment in flowing H_2_; after 30 min of treatment, the *E*_g_ mode shifted to 152 cm^−1^, indicating the removal of O sites to form oxygen vacancies, with changes in the Ti–O–Ti symmetry. For comparison, any shift of the *E*_g_ mode was undetectable with the sample in flowing argon (Supplementary Fig. 12a). And, notably, for Au/TiO_2_ in flowing H_2_, the shift of the *E*_g_ mode was slight (<4 cm^−1^) (Supplementary Fig. 11b). Similar phenomena were also indicated by the in situ Raman spectra of Pt/Sn–TiO_2_-123 (Supplementary Fig. 13). The removal of O sites from Au/Sn–TiO_2_-123 and Pt/Sn–TiO_2_-123 was confirmed by the mass signal of D_2_O in the effluent gas observed for the sample treated in flowing D_2_ (Supplementary Figs 14 and 15). When nitrobenzene was introduced to the H_2_-treated Au/Sn–TiO_2_-123 or Pt/Sn–TiO_2_-123 (Supplementary Figs 11a and 13a), the *E*_g_ mode shifted back to about 141–143 cm^−1^, associated with interaction between oxygen atoms of nitro groups with the Sn–TiO_2_ matrix—all in good agreement with the EXAFS results.

Furthermore, we emphasize that the single-site Sn is crucial for the formation of oxygen vacancies. When bulk SnO_2_ crystals were present instead of single-site Sn (in Au/Sn–TiO_2_-20), rare oxygen vacancies were formed, as confirmed by the in situ Raman spectra (Supplementary Fig. 16), D_2_-reduction (Supplementary Fig. 17), and H_2_-temperature programmed reduction (TPR, Supplementary Fig. 18) measurements, thus leading to a significant decrease in the conversion of nitrobenzene (Table 2). Further decreases in the Sn loading of Au/Sn–TiO_2_-123 led to decreased catalytic activities (Supplementary Fig. 19), because of the decreased number of oxygen vacancies in the catalysts, as confirmed by the D_2_-reduction measurements (Supplementary Fig. 20).

Moreover, we recorded IR spectra of nitrobenzene adsorbed on Au/Sn–TiO_2_-123 to better understand the catalyst–support interactions. The as-synthesized Au/Sn–TiO_2_-123 is characterized by weak bands at 1525 and 1346 cm^−1^ (Supplementary Fig. 21a), assigned to an asymmetric stretching (*ν*_asym_) vibration of nitro groups adsorbed on Ti–OH groups and a symmetric stretching (*ν*_sym_) vibration of nitro groups adsorbed on surface Ti atoms, respectively^14,32,33^. In a temperature-programmed desorption at 373 K, these bands disappeared, indicating the weakness of the interactions between nitrobenzene and Au/Sn–TiO_2_-123. Significantly, when nitrobenzene was adsorbed on H_2_-treated Au/Sn–TiO_2_-123, two bands again appeared, at 1525 and 1349 cm^−1^ (Supplementary Fig. 21b), accompanied by a band at 1493 cm^−1^, with a significant red shift from the conventional asymmetric stretching *ν*_asym_) vibration of nitro groups *ν*_asym_ (1525 cm^−1^). The red shift indicates that the N=O bonds were significantly weakened to form nitroso group by interaction with the H_2_-treated Au/Sn–TiO_2_-123 catalyst. Even after desorption at 523 K, the Au/Sn–TiO_2_-123 still exhibited a band at 1489 cm^−1^, indicating that nitro or nitroso groups were adsorbed on the catalyst. Similar phenomena were also observed in experiments with the H_2_-treated Pt/Sn–TiO_2_-123 (Supplementary Fig. 22), in agreement with the results characterizing the Au/Sn–TiO_2_-123 catalyst. Moreover, our data show that the adsorption of nitro groups on the Sn-modified catalyst was selective, hindering the adsorption of other groups (e.g., vinyl groups), as illustrated, for example, by IR spectra demonstrating that from a mixture of nitrobenzene and styrene, the former was strongly adsorbed, excluding the adsorption of the latter, thus leading to enhanced catalytic selectivity for the formation of vinyl aniline in the hydrogenation (Supplementary Fig. 23 and Supplementary Table 7).

**Fig. 2:**
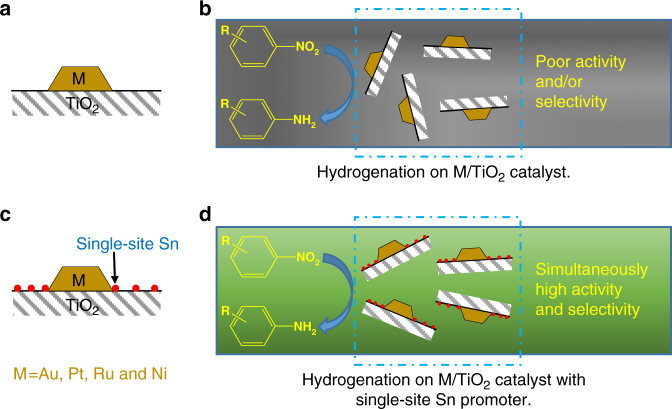
Model of catalysts with and without single-site Sn promotion. **a** Conventional M/TiO_2_ catalysts; **b** M/TiO_2_ catalysts in hydrogenation of substituted nitroarenes, for which poor activity or selectivity was obtained; **c** M/TiO_2_ catalysts with single-site Sn promoters; **d** M/TiO_2_ catalysts in hydrogenation of substituted nitroarenes, for which simultaneously high activity and selectivity were achieved by single-site Sn promotion

Taking these results together with the in situ EXAFS, Raman, and IR spectra, we infer that the adsorption/interaction is associated with the presence of oxygen vacancies near the single Sn sites on Au/Sn–TiO_2_-123. At the same time, the reactant hydrogen dissolved in or adsorbed on the metal nanoparticles readily facilitates the efficient and selective activation of nitro groups on the oxygen vacancies, which must be present near the metal nanoparticles—with the reaction taking place at the nanoparticle/support interface. These results confirm the importance of the support in Sn–TiO_2_-123 and the new strategy for selective hydrogenation of substituted nitroarenes by employing single-site Sn promotion (Fig. 2).

### Catalyst recycle

These catalysts are reusable. After each reaction experiment, the catalysts were easily separated from the reaction liquid, and, after simple washing, the catalyst was reused. In seven runs in the hydrogenation of 2-chloro-4-nitrophenol, the Au/Sn–TiO_2_-123 exhibited 2-chloro-4-nitrophenol conversions and 2-chloro-4-amino phenol selectivities that were unchanged within error (Supplementary Fig. 24), indicating the good stability. Inductively coupled plasma (ICP)-optical emission spectrometry (OES) analysis of the reaction liquid after separation of the solid catalysts gave no evidence of detectable tin or gold species, indicating the absence of measurable leaching during the reaction. The high activity and selectivity combined with the good stability make these catalysts with single Sn promoter sites potentially valuable for application.

### Density functional theory calculations

To gain deeper insight into the promotional effects of single-site Sn, we performed periodic density functional theory (DFT) calculations. It has been widely accepted that during the hydrogenation, nitrobenzene undergoes multistep reduction, successively producing nitrosobenzene, hydroxyaniline, and aniline. Our calculations demonstrate that when Sn is absent, the activity as well as the selectivity critically depend on the adsorption of nitrobenzene (Supplementary Figs 25 and 26, Supplementary Table 8). The adsorption energy of nitrobenzene on gold is rather weak (−0.04 ~ −0.27 eV), accounting for the low hydrogenation activity observed for the Au/TiO_2_ catalysts. In contrast, nitrobenzene adsorbed flat on the Pt surfaces with its phenyl group (and potential substituent groups) strongly interacting with the Pt atoms (−1.05 eV ~ −2.10 eV). Strong adsorption could explain the high hydrogenation activity of Pt/TiO_2_, and the parallel orientation of the aromatic ring could explain the low selectivity, because other substituent group could also be hydrogenated. Moreover, we found that the reduced intermediates and products would bond to the surfaces via their N atoms rather than their phenyl groups, suggesting that the hydrogenation would favor the formation of aniline once nitrosobenzene had formed. Thus, to enhance the selective hydrogenation, the promoter should facilitate the reduction of nitrobenzene to nitrosobenzene.

**Fig. 3:**
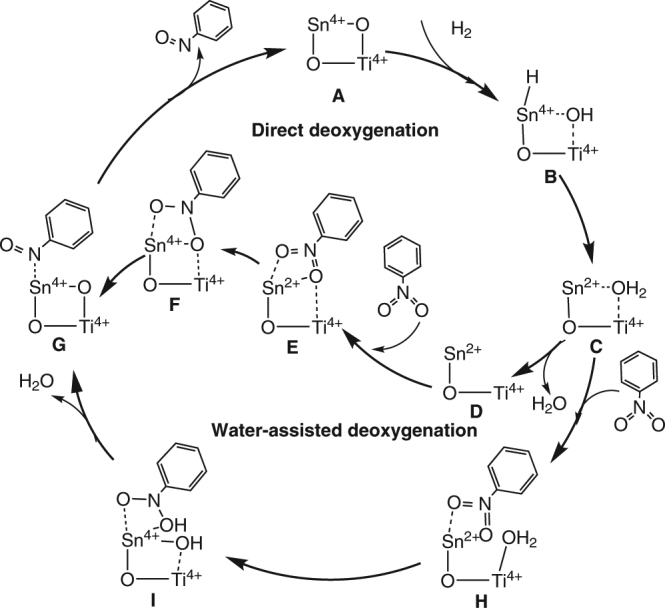
Catalytic cycle. Proposed mechanism of catalytic deoxygenation of nitrobenzene on single Sn-substituted TiO_2_ surfaces

The calculations show that single-site Sn favors the generation of oxygen vacancies under the hydrogenation conditions (Supplementary Table 9), in agreement with the experimental results. Figure 1 shows the potential energy profile of a complete catalytic cycle on Sn_1_/TiO_2_(101) (Fig. 3 and Supplementary Fig. 27). H_2_ activated heterolytically on the Sn^4+^–O sites forms Sn(–H)–OH species (**B**), followed by H transfer to form an oxygen vacancy and adsorbed H_2_O (**C**).

For the reduction of nitrobenzene, we considered two possible mechanisms, direct deoxygenation and water-assisted deoxygenation (Figs 1 and 3). In the former case, two adsorption states of nitrobenzene (**E** and **F**) on the oxygen vacancy were identified, with the interactions with the surface via the nitro groups rather than phenyl group, in contrast to the chemistry that occurs on noble metal surfaces^14,34,35^. Estimates of the Bader charges show that the nitrobenzene moiety in species **E** is nearly neutral (−0.02 a.u.), whereas in **F**, the nitrobenzene carries a charge of −0.56 a.u.—thus, **E** represents only physisorption whereas **F** represents a charge-transfer state. The computations show that a conversion of **E** to **F** requires overcoming a barrier of 0.95 eV (**TS**_**E-F**_), whereas that from **F** to **G**, the nitrosobenzene adsorption state, requires overcoming a barrier of only 0.23 eV (**TS**_**F-G**_).

For water-assisted deoxygenation, the adsorbed H_2_O from the reaction was directly bound with Ti^4+^, creating acidic groups. As shown in Fig. 1, nitrobenzene could be trapped by the adsorbed H_2_O though an OH···ONO hydrogen-bonding interaction, forming a physically adsorbed species (**H**). Next, **H** would undergo hydrogen transfer to give **I** (**TS**_**H-I**_). Although **H** and **I** were found to be nearly the same in energy (−1.41 vs. −1.42 eV), they have different electronic configurations. Through charge analysis, we found that Sn still maintained the oxidation state of + 2 in **H**, whereas Sn in **I** had been oxidized to Sn^4+^. Thus, **TS**_**H-I**_ can be viewed as having been formed by a proton coupling electron transfer in which electrons transfer from Sn to N, while a proton is transferred from H_2_O to the nitro group. In contrast to the formation of **TS**_**E-F**,_ the formation of **TS**_**H-I**_ involves only a small barrier of 0.25 eV. This mechanism can be rationalized by considering that the adsorbed H_2_O stabilizes the negatively charged nitrobenzene during the electron transfer. In the next step, by overcoming a barrier of 0.20 eV, **I** would undergo another hydrogen transfer to produce adsorbed nitrosobenzene and reform the oxygen vacancy (**G**).

## Discussion

The combination of experimental and theoretical results leads to a clear picture of this new class of catalyst and its multiple functions. The theoretical results, consistent with experiment, indicate that the water-assisted deoxygenation mechanism is favored over the direct deoxygenation (Supplementary Figs 28 and 29). The selective hydrogenation of nitrobenzene thus benefits from the complementary roles of the catalyst components—the site-isolated Sn cations on the anatase support; the oxygen vacancies formed near these sites where the nitro groups are adsorbed; the nearby metal nanoparticles where reactant hydrogen is activated and made available to readily facilitate the generation of oxygen vacancies. These catalyst components act in concert and must be nearby each other, engaged in a mechanism whereby Sn_1_/TiO_2_ facilitates the deoxygenation of nitrobenzene to nitrosobenzene, whereas the noble metal, in addition to facilitating H_2_ dissociation, provides the sites for hydrogenation of nitrosobenzene intermediate to give aniline. We emphasize that the specific interaction between the Sn_1_/TiO_2_ support with its oxygen vacancies and reactant nitro groups is essential for the high activity and selectivity in the hydrogenation of nitroarenes on Sn–TiO_2_-123 and the extended family of supported metal catalysts.

In summary, we present a strategy for improving the catalytic performance of a range of metal catalysts on conventional TiO_2_ supports by employing single-site Sn sites on the TiO_2_ as promoters. Gold, platinum, ruthenium, and nickel nanoparticles on the promoted support exhibit enhanced catalytic activity and selectivity for the hydrogenation of various substituted nitroarenes. Multi-technique characterization of the catalysts showed that the single-site Sn species are associated with oxygen vacancies formed in an H_2_ atmosphere that facilitate selective adsorption and activation of nitro groups. The single-site Sn-promoted catalysts are stable and highly active and selective. The strategy of employing single-site promoters for selective supported metal catalysts may open the way to other catalysts with comparable combinations of components.

## Methods

### Catalyst synthesis

Synthesis of Sn–TiO_2_-123: In a typical experiment, 2.0 g of commercial anatase (20–50 nm) was initially heated at 100 °C for 10 h under vacuum in a 100 mL flask. After the addition of anhydrous toluene (60 mL, dried by P_2_O_5_) and dimethyltin dichloride (69 mg), the mixture was stirred at room temperature for 0.5 h. Then 5 mL of triethylamine was added, and the mixture was continuously stirred for another 2 h at room temperature. After filtration, washing with toluene and ethanol, drying, and treatment at 580 °C for 3 h in air, Sn–TiO_2_-123 was finally obtained. The Ti/Sn atomic ratio of Sn–TiO_2_-123 was 123. By increasing the amount of dimethyltin dichloride, Sn–TiO_2_-20 was obtained. By using SiO_2_ instead of TiO_2_ as a support, Sn–SiO_2_-129 with a Si/Sn ratio of 129 was obtained. By using 425 mg of dimethyltin dichloride in the starting solution, Sn–TiO_2_-20 was obtained following the same procedures.

Synthesis of the supported Au and Pt catalysts: The supported metal catalysts were synthesized by the deposition-precipitation method. In a typical experiment for the synthesis of Au/Sn–TiO_2_-123, 0.5 g of Sn–TiO_2_-123 was added to 100 mL of HAuCl_4_ (0.028 mmol of Au) and urea (2.9 mmol) solution. After stirring at 363 K for 4 h in a closed reactor kept in the dark, the liquid mixture was cooled in an ice bath at 273 K, followed by addition of NaBH_4_ solution (400 mg of NaBH_4_ in 20 mL of water). Finally, the solid sample was filtered and washed with a large amount of water, dried at 373 K for 12 h, and calcined at 473 K for 4 h. The Au/Sn–TiO_2_-123 sample was then obtained.

### Catalytic reaction experiments

The hydrogenation reactions were performed in a high-pressure autoclave with a magnetic stirrer (1000-1200 rpm). Typically, the reactant (substrate), catalyst, and solvent were mixed in the reactor and stirred for 15 min at room temperature. Then pure H_2_ was introduced and kept at a desired pressure (measured at the reaction temperature), and the reaction system was heated to a given temperature (measured with a thermometer in the autoclave). After the reaction, the solid catalyst was separated from the fluid products, and the products were analyzed by gas chromatography (GC-14C, Shimadzu, with a flame ionization detector) with a flexible quartz capillary column coated with OV-17 or FFAP. The recyclability (reusability) of the catalyst was tested by separating it from the reactor liquid contents by centrifugation, washing with a large volume of methanol, and drying at 353 K for 6 h. In the recyclability test of Au/Sn–TiO_2_-123, the gold concentration in the mother liquor was found to be less than 5 ppb by ICP analysis after separation of the used solid catalyst, indicating that essentially no gold leaching occurred during the reaction.

### Catalyst characterization

X-ray photoelectron spectra were recorded with a Thermo ESCALAB 250 with Al Kα radiation at *h* = 90° for the X-ray sources; the binding energies were calibrated by using the C1s peak at 284.9 eV. IR spectra were recorded with a Nicolet NEXUS 670 FT-IR spectrometer equipped with an MCT detector and ZeSe windows and a high-temperature reaction chamber. The nitrobenzene stream was introduced into the system in a flow of argon carrier gas. Raman spectra were recorded using a HR800 Raman spectrometer equipped with an Ar excitation source (λ = 514.532 nm). The nitrobenzene stream was introduced into the system with a flow of Ar carrier gas (30 sccm) with the reactant partial pressure in the range of 10–20 mBar. The catalyst metal content was determined by ICP analysis with a Perkin–Elmer Plasma 40 emission spectrometer and X-ray fluorescence. High-resolution transmission electron microscopy imaging was carried out with a FEI-Titan ST electron microscope operated at 300 kV with a point resolution of 0.19 nm; the Au nanoparticle size distribution was determined by counting more than 100 nanoparticles.

Mass spectrometry: Mass spectra of the effluent gases introduced into a flow system or produced by reaction with the sample were measured with an online Balzers OmniStar mass spectrometer running in multi-ion monitoring mode. The sample was pressed into a thin wafer and loaded into a cell (In-situ Research Institute, South Bend, IN) through which helium continuously flowed at a rate of 100 mL/min. After the temperature had been increased to reach 393 K, D_2_ was added to the helium stream with a flow rate of 20 mL/min. Changes in the intensities of major fragment of D_2_O (*m*/*z* = 20) were recorded. A bypass was used to determine whether D_2_O was formed in the sample. During the bypass, D_2_ and helium flowed directly to the mass spectrometer without coming in contact with the sample. After switching the flow back to the line allowing gas to flow through the cell, a sharp increase in the D_2_O signal was observed, indicating that D_2_O was formed from the sample in the D_2_-containing environment.

X-ray absorption spectroscopy: The X-ray absorption spectra were collected at beamline 4-1 at the Stanford Synchrotron Radiation Lightsource and at beamline (BL14W1) at the Shanghai Synchrotron Radiation Facility. A Si (220) or Si(111) double crystal monochromator was used and was detuned to 70% of maximum intensity to reduce the interference of higher harmonics present in the X-ray beam. The sample (approximately 0.6 g) was loaded into a vacuum sample holder and cooled to liquid nitrogen temperature during the measurement. For operando EXAFS spectroscopy, the sample was loaded into a flow-through cell and then was treated in flowing H_2_ (10% H_2_/He, flow rate at 20 sccm) or nitrobenzene (He flow through the liquid nitrobenzene at 353 K, flow rate at 20 sccm) at 373 K. The data were collected in transmission mode by use of ion chambers mounted on each end of the sample holder. A tin foil was placed down the beam of the second ion chamber and simultaneously measured as a reference.

The methods for data analysis of X-ray absorption spectroscopy and DFT calculations are available in the Supplementary Methods.

### Data availability

All data are available within the article and its Supplementary Information file or from the authors upon request.

## Electronic supplementary material

Peer Review File

Supplementary Information
