# Supplementary material for: Single-site catalyst promoters accelerate metal-catalyzed nitroarene hydrogenation
Source: Nat Commun. 2018 Apr 10;9:1362. doi: 10.1038/s41467-018-03810-y (PMC5893533; doi:10.1038/s41467-018-03810-y)
Supplement: Supplementary file 2 — Supplementary Information [file 41467_2018_3810_MOESM2_ESM.docx]

**Supplementary Information**

**Single-Site Catalyst Promoters Accelerate**

**Metal-Catalyzed Nitroarene Hydrogenation**

**Wang et al.**

**Supplementary methods**

**Chemicals.** All reagents were of analytical grade and used as purchased without further purification.

**Synthesis of the supported Ru and Ni catalysts.** In a typical experiment, 0.5 g of Sn-TiO_2_-123 was added to 100 mL of ruthenium chloride or nickel nitrate solution. After ultrasonic treatment for 30 min, urea was added (mole ratio of urea/metal was 130), the mixture was stirred at room temperature for another 2 h. Then the water was removed under vacuum, the solid powder was heated at 373 K for 24 h, calcined at 673 K for 4 h in air, and reduced in H_2_ (473 K, 4 h for Ru catalyst and 623 K, 6 h for Ni catalyst) to obtain the final catalyst.

**XANES and EXAFS Data Analysis.** Analysis of the X-ray Absorption Near Edge Structure (XANES) and Extended X-ray Absorption Fine Structure (EXAFS) data was carried out with the software ATHENA of the IFEFFIT package^1,2^, and the EXAFS data were further analyzed with the software XDAP developed by Vaarkamp *et al.*^3^. Three spectra were averaged to give the spectrum that was subjected to analysis. Details of the data fitting methodology are reported elsewhere^4,5^.

The error in the data was calculated as the root mean square of the value obtained from the subtraction of smoothed χ (χ is the EXAFS function) data from the background-subtracted experimental χ values. Goodness of fit values were calculated with the software XDAP, as follows:

$$goodness of fit= \frac{\nu}{NPTS(\nu-N_{\mathrm{free}})}\sum_{i=1}^{\mathrm{NPTS}} (\frac{\chi_{exp,i}-\chi_{model,i}}{\sigma_{exp,i}})^{2} (1)$$

In this equation, *χ_model_* and *χ_exp_* are the model and experimental EXAFS values; *σ_exp_* is the error in the experimental results; ν is the number of independent data points in the fit range; and NPTS is the actual number of data points in the fit range; *N*_free_ is the number of free parameters.

Several models were fitted to the data and compared with each other on the basis of the goodness of fit and the quality of the overall fit in both *k* space and *R* space. A "difference-file" technique was applied to the candidate models, whereby the calculated EXAFS contribution from each individual Sn-backscatterer contribution was compared with the data in *R* space (calculated by subtracting all the other calculated Sn-backscatterer contributions from the experimental overall contributions). The best-fit model is the one providing optimum agreement between the model and the calculated EXAFS data in *k* space, *R* space, and for individual shells.

Note that the results characterizing the as-synthesized Au/Sn-TiO_2_-123 shown in Table 1 are from an independent EXAFS experiment—different from that represented by the data in Supplementary Figure 12. The results from two independent experiments are consistent with each other with regard to both the refined Sn-O/Sn-Ti coordination number and bond distance.

**DFT Calculations.** All calculations were performed by using density functional theory (DFT) as implemented in the Vienna *ab-initio* simulation package (VASP5.3.5)^6-9^. Exchange and correlation potentials were treated within the Perdew-Burke-Ernzerhof (PBE) functional^10^. The valence electrons were described by plane wave basis sets with a kinetic energy cut-off of 400 eV, and the core electrons were replaced by the projector augmented wave pseudopotentials ^11,12^.

To model the lower Miller index surfaces of noble metals, such as Au and Pt, five-layer slabs were used. To prevent periodic image interactions, we adopted (4 x 3), (3 x 3) and (3 x 3) supercell for the (111), (100), and (110) surfaces, respectively. For anatase TiO_2_ surfaces, the major exposed facets, such as (101), (100), and (001), were considered, and the p(3x3), p(3 x 1) and p(3 x 3) supercells with five-layer slabs were adopted, respectively. Because the substitutional solid solutions Sn_x_Ti_1-x_O_2_ with a large range of x could be formed, as shown experimentally^13^, we expected that the Sn atom doping would displace a Ti site on anatase TiO_2_ surfaces, resulting in Sn_1_/TiO_2_ models. For simplicity, in each TiO_2_ surface model, only one surface 5-coordinated Ti was substituted by Sn. During structural optimization, the bottom two slab layers were fixed at their bulk positions, while the top three layer slabs and the adsorbates were allowed to be fully relaxed. In all simulations, a vacuum gap of 15 Å separated the periodic images of the slab along the c direction and Monkhurst-Pack *k*-point sampling with approximately 0.05 × 2π Å^-1^ spacing in a reciprocal lattice was used.

The minimum energy reaction pathways were calculated by using the nudged elastic band method. The final transition state structures were refined by using the quasi-Newton algorithm until the Hellman-Feynman forces on each ion were lower than 0.05 eV/Å. Frequency calculations were performed to confirm each transition state had only one imaginary frequency. The adsorption energies (Δ*E*) were calculated by using Equation (2), in which *E*_ad_/sub, *E*_ad_, and *E*_sub_ were the total energies of the optimized adsorbate/surface system, the adsorbate in the gas phase, and the clean surface, respectively.

Δ*E* = *E*_ad_/_surf_ – *E*_ad_ – *E*_surf_ (2)

Because O_2_ and O atoms are poorly described by PBE functionals, we used the Δ*E*_HDO_ values to evaluate the binding ability of O atoms, defined as the reaction energy of the following reaction:

X-O + H_2_ → X + H_2_O (3)

Δ*E*_HDO_ = *E*(X) + *E*(H_2_O) - *E*(XO) - *E*(H_2_) (4)

Here, X-O are the substrates or oxides. The higher the value of Δ*E*_HDO_, the greater the ability of X to bind an O atom, and the lower the probability of generating the oxygen vacancy. Thus, to be a good catalyst, the oxide should have an appropriate value of Δ*E*_HDO_ which would be less than zero and greater than that of substrate.

To estimate the formal oxidation state of Sn, we used the Bader charge analysis. Bulk SnO_2_ with the rutile structure and bulk SnO with the litharge structure were adopted as references. According to our calculations, the Bader charge of Sn in Sn(IV)O_2_ and Sn(II)O were 2.38 a.u. and 1.23 a.u., respectively.


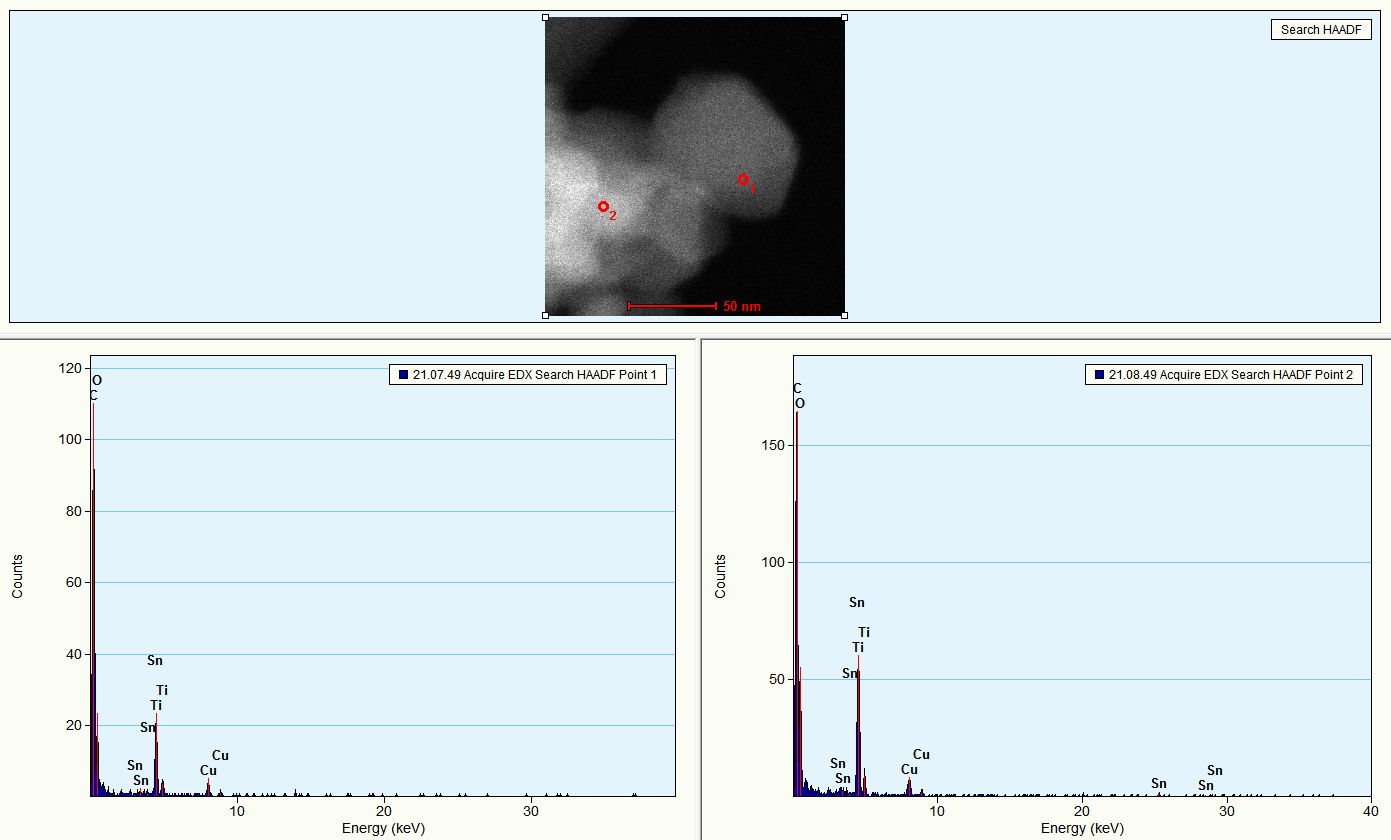

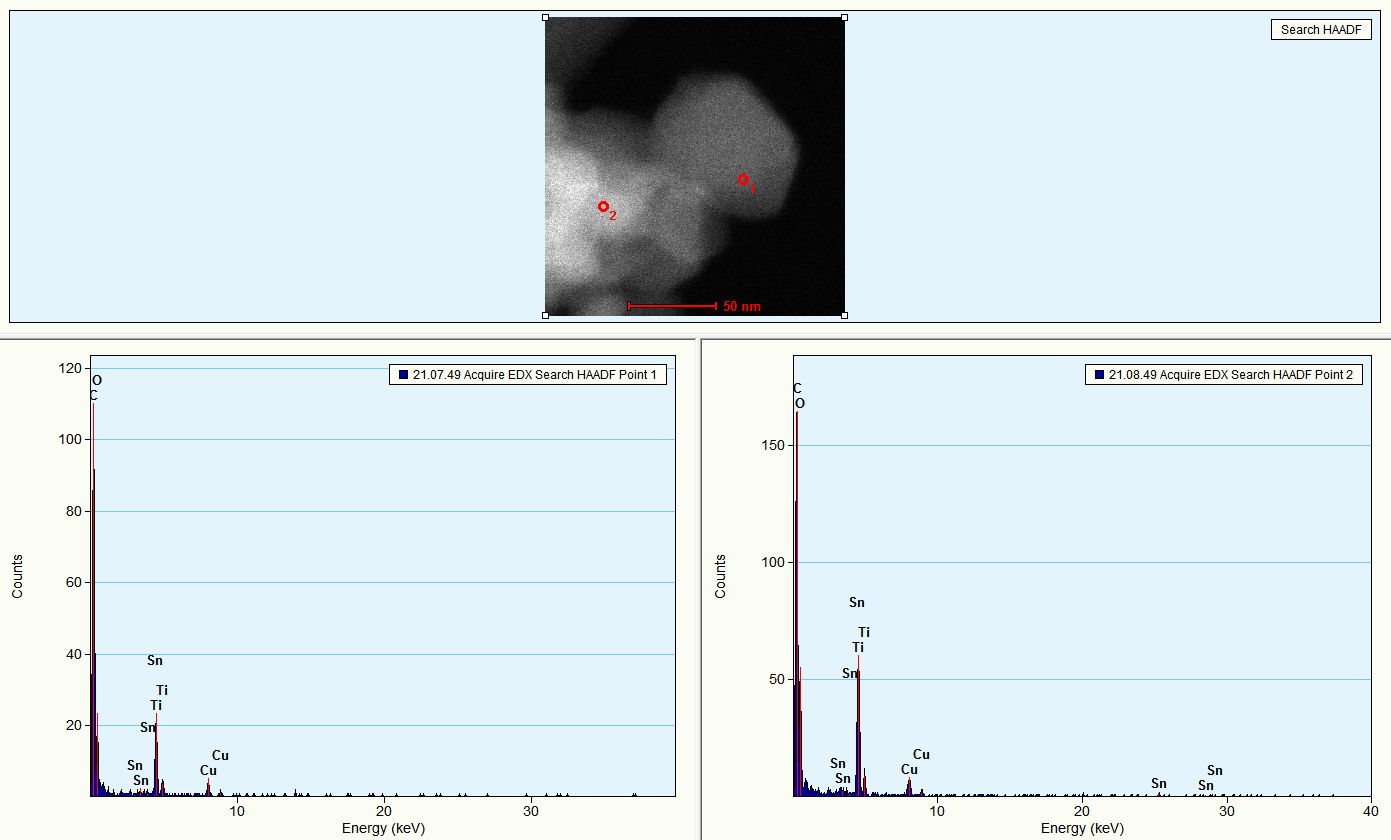


Region 1

Region 2

**Supplementary Figure 1.** EDX analysis of randomly selected regions of Sn-TiO_2_-123 sample.

TiO_2_(103)

Au(111)

SnO_2_

(110)

(b)

(a)

SnO_2_

(101)

**Supplementary Figure 2.** (a) XRD patterns and (b) Sn3d XP spectra of (a) Au/TiO_2_, (b) Sn-TiO_2_-123, and (c) Au/Sn-TiO_2_-123.

**Note:** Supplementary Figure 2a shows the XRD pattern of Au/Sn-TiO_2_-123, assigned to a typical anatase structure. Peaks that would be associated with metallic gold or SnO_2_ are absent, indicating the high dispersion of the gold and tin species. Sn3d X-ray photoelectron spectra (XPS) of Sn-TiO_2_-123 and Au/Sn-TiO_2_-123 (Supplementary Figure 2b) both exhibit Sn3d peaks with similar binding energies, indicating that the tin binding energy was not changed as a result of loading of gold nanoparticles).


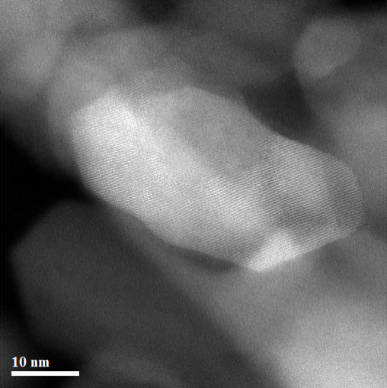

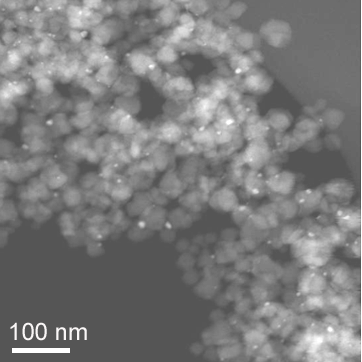


(a)

(b)

(c)


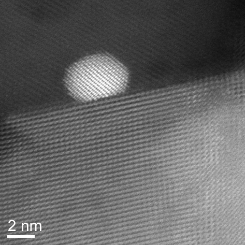


**Au**

**d=0.239 nm**

**TiO_2_**

**Supplementary Figure 3.** (a) HAADF-STEM image of Au/Sn-TiO_2_-123. Inset: gold nanoparticle size distribution. High-resolution TEM images of (b) Au/Sn-TiO_2_-123 and (c) Sn-TiO_2_-123.


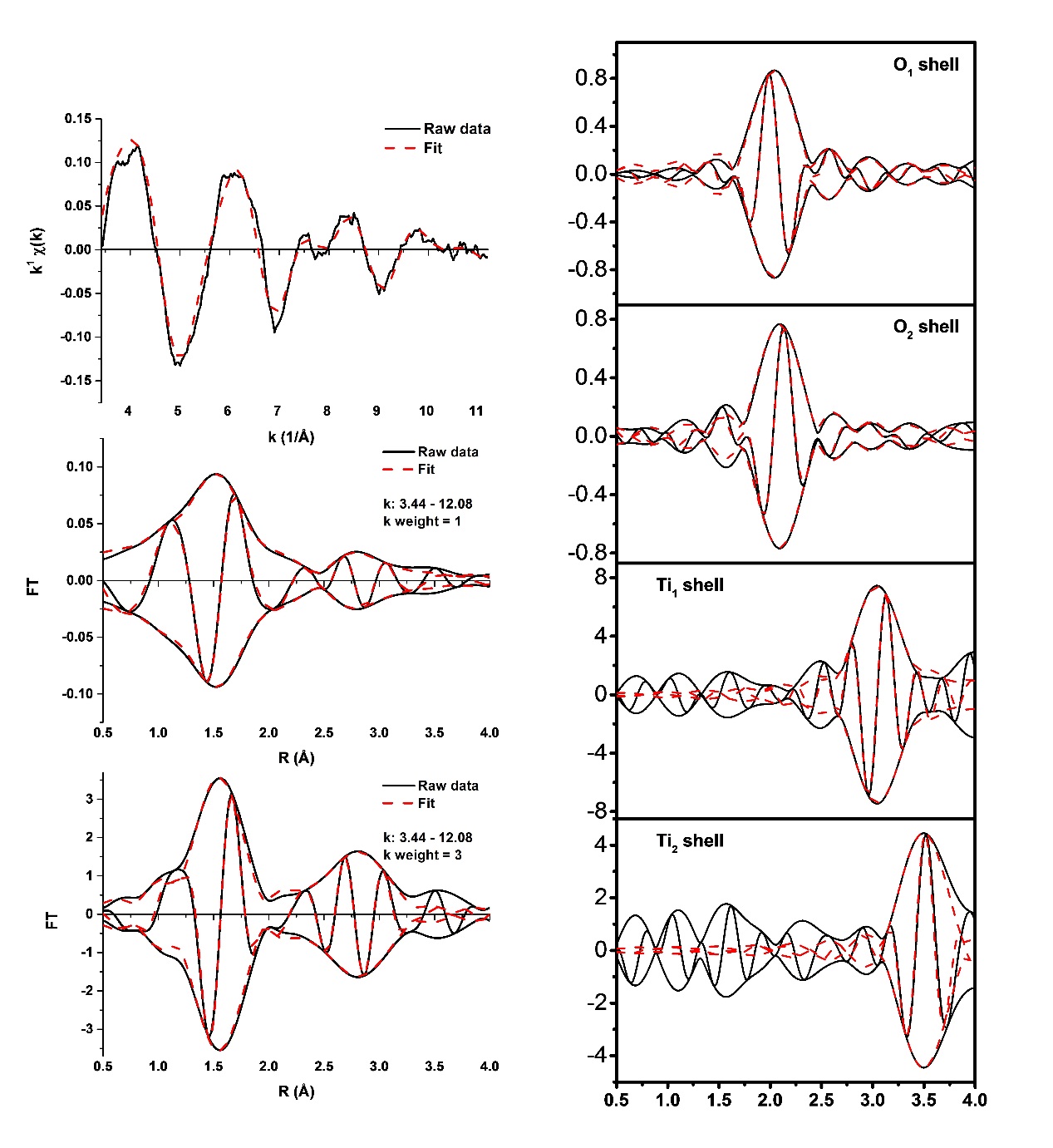


**R (Å)**

**Supplementary Figure 4.** EXAFS data characterizing Sn-TiO_2_-123: **k**^1^-weighted EXAFS function, **k**^1^(χ) (solid line) and sum of the calculated contributions (dashed line); **k**^1^-and **k**^3^-weighted and single-shell imaginary part and magnitude of the Fourier transform of the data (solid line) and sum of the calculated contributions (dashed line) of the samples represented in Table 1.

**Supplementary Figure 5.** XANES data characterizing tin foil, Au/Sn-TiO_2_-123, and SnO_2_. The reference data of Sn and SnO_2_ samples are from Refs. 15 and 16, respectively.

**Supplementary Table 1.** Edge position of tin foil, Au/Sn-TiO_2_-123, and SnO_2_.

| **Sample** | **Measured edge position (eV)** | **Reported edge positions (eV)** |
| --- | --- | --- |
| Sn | 29200 | 29200 |
| Au/Sn-TiO_2_-123 | 29205 | This work |
| SnO_2_ | 29208 | 29209 |


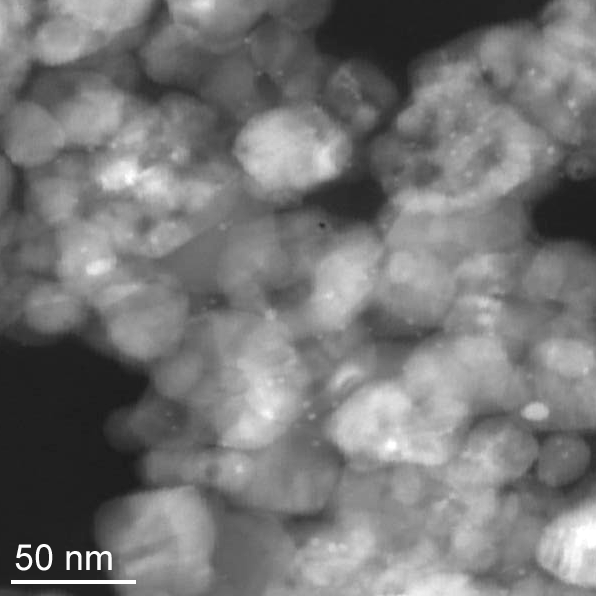


**Supplementary Figure 6.** TEM image and gold nanoparticle size distribution of Au/TiO_2_. Inset: gold nanoparticle size diameter distribution.

**Supplementary Figure 7.** XRD pattern of Au/Sn-TiO_2_-20. The peak of SnO_2_ appeared in the XRD pattern, indicating the presence of bulky SnO_2_ particles.


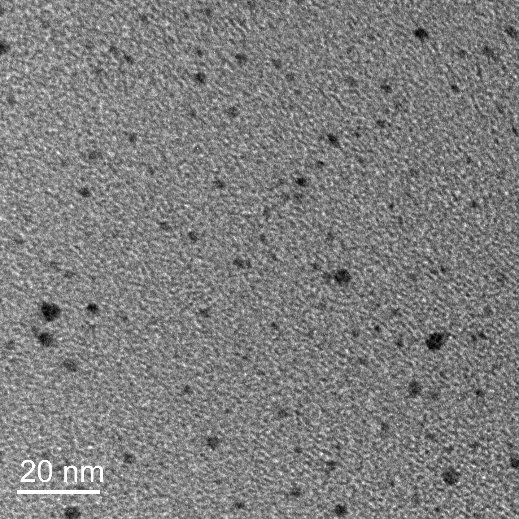


**(b)**


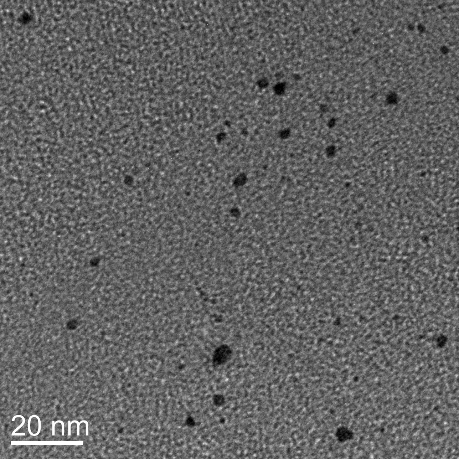


**(a)**

**Supplementary Figure 8.** TEM images of (a) Au/SiO_2_ and (b) Au/Sn-SiO_2_-129.

**Supplementary Figure 9.** XRD pattern of Au/Sn-SiO_2_-129.

**R (Å)**

**R (Å)**

**Supplementary Figure 10.** EXAFS data characterizing Au/Sn-TiO_2_-123 under *in-situ* treatment conditions: **k**^3^-weighted and single-shell imaginary part and magnitude of the Fourier transform of the data (solid line) and sum of the calculated contributions (dashed line) of the samples represented in Table 1 in the main text.

(a)

(b)

**Supplementary Figure 11.** Raman spectra of (a) Au/Sn-TiO_2_-123 and (b) Au/TiO_2_ under *in-situ* treatment conditions with tandem steps of H_2_ treatment at 373 K for (a) 0, (b) 20, (c) 40, and (d) 60 min; nitrobenzene treatment at 373 K for (e) 20, (f) 40 min, and argon treatment at 573 K for (g) 20, (h) 40, and (i) 60 min.

(a)

(b)

**Supplementary Figure 12.** *In-situ* Raman spectra of (a) Au/Sn-TiO_2_-123 and (b) Sn-TiO_2_-123 under *in-situ* treatment conditions with tandem steps of argon treatment (for Au/Sn-TiO_2_-123) or H_2_ treatment (for Sn-TiO_2_-123) at 373 K for (a) 0, (b) 20, (c) 30, (d) 40, and (e) 60 min, nitrobenzene treatment at 373 K for (f) 20, (g) 30, and (h) 40 min, and argon treatment at 473 K for (i) 20 and (j) 60 min.

(a)

(b)

**Supplementary Figure 13.** Raman spectra of various samples under *in-situ* treatment conditions with tandem steps. (a) Pt/Sn-TiO_2_-123 under H_2_ treatment conditions at 343 K for (a) 0 and (b) 30 min, (c) nitrobenzene treatment conditions at 343 K for 30 min, and (d) argon treatment at 473 K for 60 min. (b) Pt/TiO_2_ under H_2_ treatment at 343 K for (a) 0, (b) 20, and (c) 40 min. The Pt/Sn-TiO_2_-123 data exhibit a markedly shifted *E_g_* mode after treatment in flowing H_2_, whereas the shift observed for Pt/TiO_2_ undergoing the equivalent treatment is very slight.

**Supplementary Figure 14.** Mass spectra of effluent gases from (a) TiO_2_, (b) Sn-TiO_2_-123, and (c) Au/Sn-TiO_2_-123 at *m*/*z* = 20 in a bypass test with flowing D_2_ in helium at 393 K. Au/Sn-TiO_2_-123 and Sn-TiO_2_-123 exhibit significant signals characteristic of D_2_O (highlighted by the arrows), indicating the efficient removal of oxygen sites by D_2_. In contrast, the D_2_O signal is very weak with TiO_2_ only.

**Supplementary Figure 15.** Mass spectra of effluent gases from (a) TiO_2_, (b) Sn-TiO_2_-123, and (c) Pt/Sn-TiO_2_-123 at *m*/*z* = 20 in a bypass test with flowing D_2_ in helium at 343 K. The spectra of TiO_2_ and Sn-TiO_2_-123 are obtained by an individual test from those in Supplementary Figure 15. Pt/Sn-TiO_2_-123 and Sn-TiO_2_-123 exhibit significant signals characteristic of D_2_O (highlighted by the arrows), indicating the efficient removal of oxygen sites by D_2_. In contrast, the D_2_O signal is very weak with TiO_2_ only.

**Supplementary Figure 16.** Raman spectra of Au/Sn-TiO_2_-20 under *in-situ* conditions of H_2_ treatment at 373 K for (a) 0, (b) 10, (c) 20, (d) 30, and (e) 40 min. The Raman spectrum of Au/Sn-TiO_2_-20 is characterized very slight shift of *E_g_* mode (≤3 cm^-1^) before and after the reduction treatment, suggesting the lack of oxygen vacancies.

**Supplementary Figure 17.** Mass spectra of effluent gases from (a) Au/Sn-TiO_2_-123 and (b) Au/Sn-TiO_2_-20 at *m*/*z* = 20 in a bypass test with flowing D_2_ in helium at 373 K. The spectrum of Au/Sn-TiO_2_-123 was obtained in an individual test from those in Supplementary Figure 15. Au/Sn-TiO_2_-123 exhibits a stronger signal characteristic of D_2_O (highlighted by the arrows) than Au/Sn-TiO_2_-20, demonstrating the efficiency in promoting oxygen vacancies on Au/Sn-TiO_2_-123.

**Supplementary Figure 18.** H_2_-TPR profiles of (a) Au/Sn-TiO_2_-123 and (b) Au/Sn-TiO_2_-20. The Au/Sn-TiO_2_-123 gives stronger signals assigned to the oxidation of H_2_ by the surface oxygen species than Au/Sn-TiO_2_-20 in the low-temperature region (350-460 K), suggesting the formation of active surface oxygen species by single-site Sn promotion.

**Supplementary Figure 19.** Dependence of reaction rate on Ti/Sn ratio in the hydrogenation of nitrobenzene on the Au/Sn-TiO_2_ catalysts with various Sn loadings. Reaction conditions: batch reactor at 373 K, 10 mmol of nitrobenzene, 8 mg of catalyst, 10 mL of toluene, 1.3 MPa of H_2_, reaction time: 15 min. The TOFs are underestimates calculated on the basis of all the Au sites in the reaction system.

**Supplementary Figure 20.** Mass spectra of effluent gases from (a) Au/Sn-TiO_2_-123, (b) Au/Sn-TiO_2_-170, (c) Au/Sn-TiO_2_-215, and (d) Au/Sn-TiO_2_-300 at *m*/*z* = 20 in a bypass test with flowing D_2_ in helium at 373 K. the intensity of D_2_O signals decreased with decreasing Sn loading indicating that Sn sites at lower loadings promote the formation of fewer oxygen vacancies.

(a)

(b)

**Supplementary Figure 21.** Nitrobenzene adsorption characterized by IR spectra of (a) as-synthesized and (b) H_2_-treated Au/Sn-TiO_2_-123 under programmed desorption conditions: (a) 323 K, 4 min, (b) 323 K, 8 min, (c) 323 K, 12 min, (d) 373 K, 4 min, (e) 373 K, 8 min, (f) 423 K, 4 min, (g) 423 K, 8 min, (h) 473 K, 4 min, (i) 473 K, 8 min, (j) 523 K, 4 min, (k) 523 K, 8 min. The band at 1590 cm^-1^ is assigned as the aromatic ring vibration frequency.

(b)

(a)

**Supplementary Figure 22.** Nitrobenzene adsorption characterized by IR spectra of (a) as-synthesized Pt/Sn-TiO_2_-123 under desorption conditions (a) 323 K, 1 min, (b) 323 K, 5 min, (c) 323 K, 10 min, (d) 373 K, 5 min, and (b) H_2_-treated Pt/Sn-TiO_2_-123 under desorption conditions (a) 323 K, 5 min, (b) 323 K, 10 min, (c) 373 K, 10 min, (d) 473 K, 10 min, (e) 523 K, 10 min.

**Supplementary Figure 23.** *Operando* IR spectra characterizing the competitive adsorption of nitrobenzene and styrene on the Pt/TiO_2_ and Pt/Sn-TiO_2_-123 catalysts. The catalysts were pre-reduced by treatment in flowing H_2_, and then a mixture of styrene and nitrobenzene was introduced. The spectra were recorded after a 10-min period for desorption (of weakly bound species) at 323 K.

We studied the competitive adsorption of a mixture of nitrobenzene and styrene on the Pt/TiO_2_ and Pt/Sn-TiO_2_-123 catalysts using *operando* IR spectroscopy. The IR spectrum of the Pt/TiO_2_ exhibits the bands associated with both nitro (1525, 1491, and 1346 cm^-1^) and vinyl groups (1417, 1447, and 1630 cm^-1^), indicating simultaneous adsorption of nitrobenzene and styrene. However, it is important that the IR spectrum of the Pt/Sn-TiO_2_-123 includes the bands associated only with the nitro group (1525, 1491, and 1346 cm^-1^), indicating the selective adsorption of nitrobenzene on the Pt/Sn-TiO_2_-123 (the band at 1590 cm^-1^ is assigned to the aromatic ring), whereby the styrene adsorption is prevented by the nitrobenzene. These results demonstrate a unique feature of the Pt/Sn-TiO_2_-123 for selective adsorption of nitrobenzene, which should be responsible for a significant enhancement of vinyl aniline selectivity in the hydrogenation of 3-nitrostyrene on the Pt/Sn-TiO_2_-123 catalyst.

**Supplementary Figure 24.** Recycle test of Au/Sn-TiO_2_-123 in the hydrogenation of 2-chloro-4-nitrophenol. The reaction conditions are the same to those stated in Table 2. The reaction time was short, 1 h.


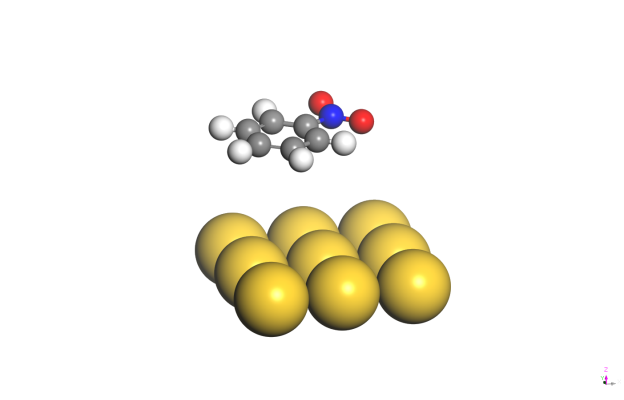

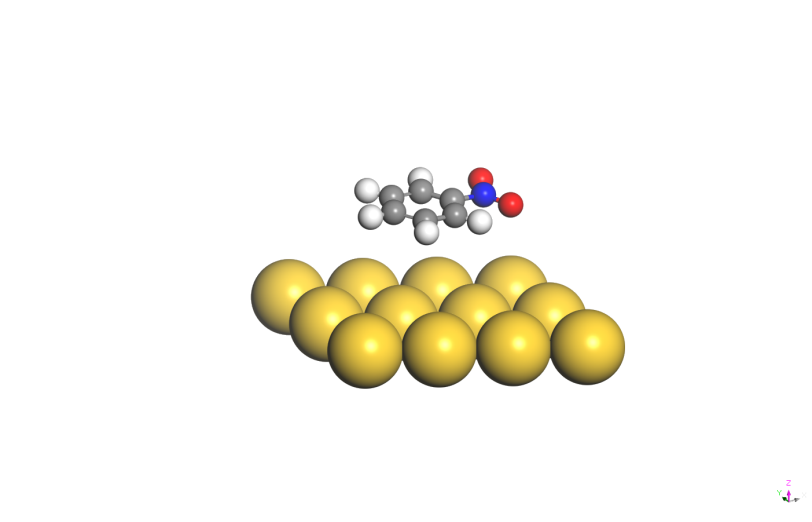

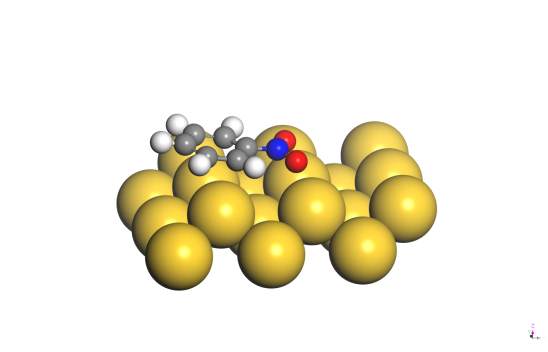

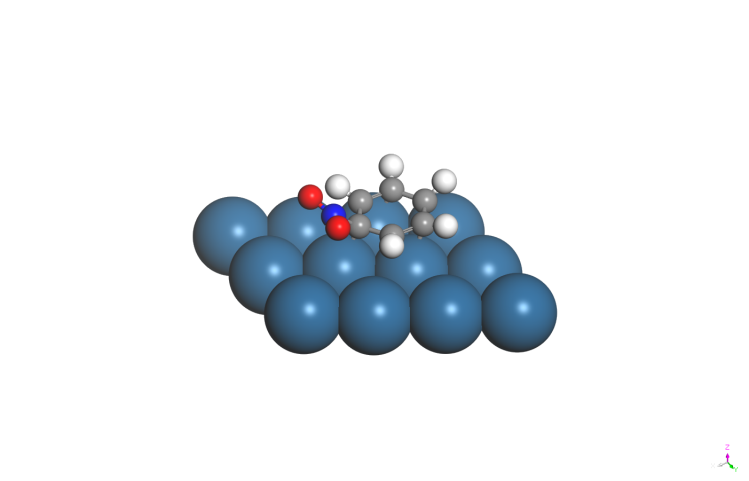

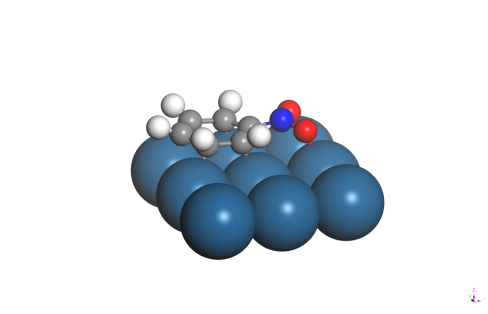

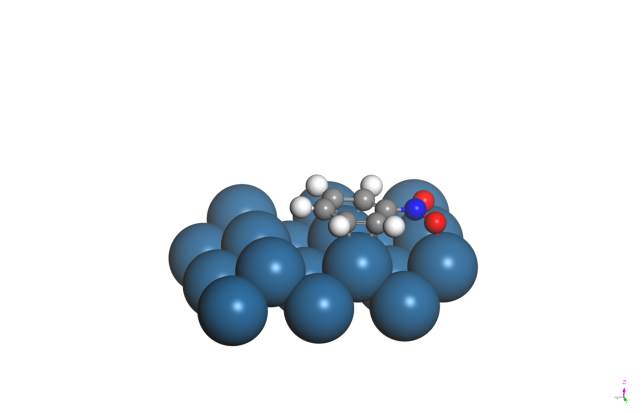


**Au(110)**

**Au(100)**

**Au(111)**

**Pt(110)**

**Pt(100)**

**Pt(111)**

**Supplementary Figure 25.** Optimized structures of nitrobenzene adsorption on Au and Pt surfaces.

**
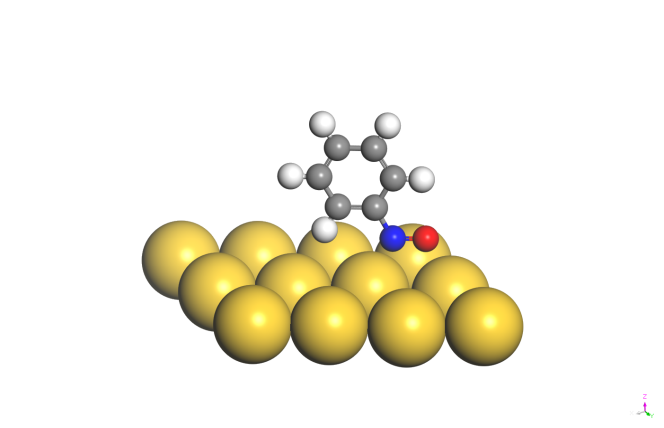

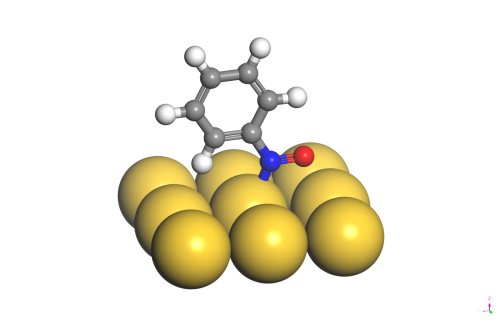

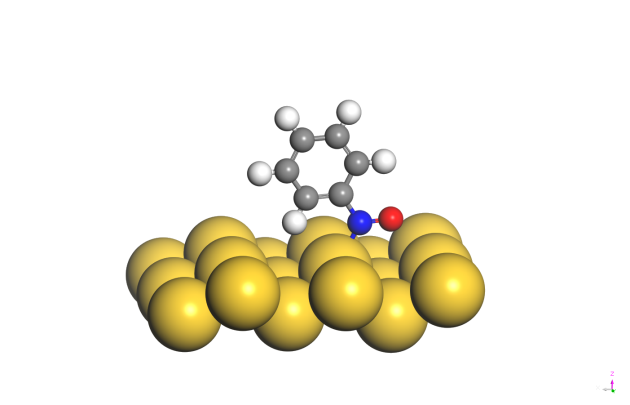

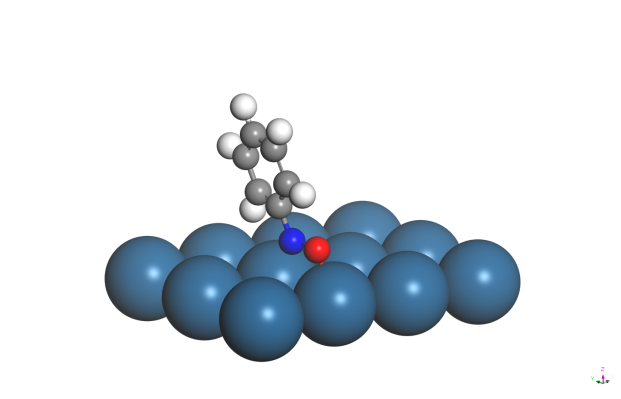

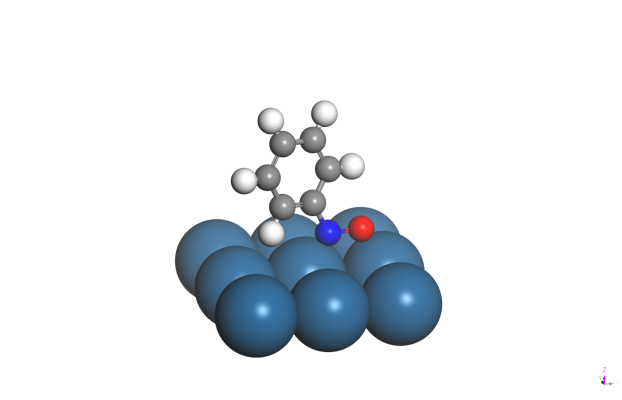

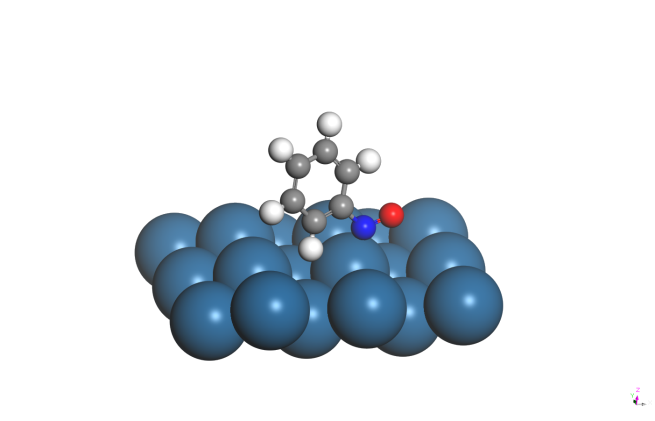
**

**Pt(110)**

**Pt(100)**

**Pt(111)**

**Au(110)**

**Au(100)**

**Au(111)**

**Supplementary Figure 26.** Optimized structures of nitrosobenzene adsorption on Au and Pt surfaces.


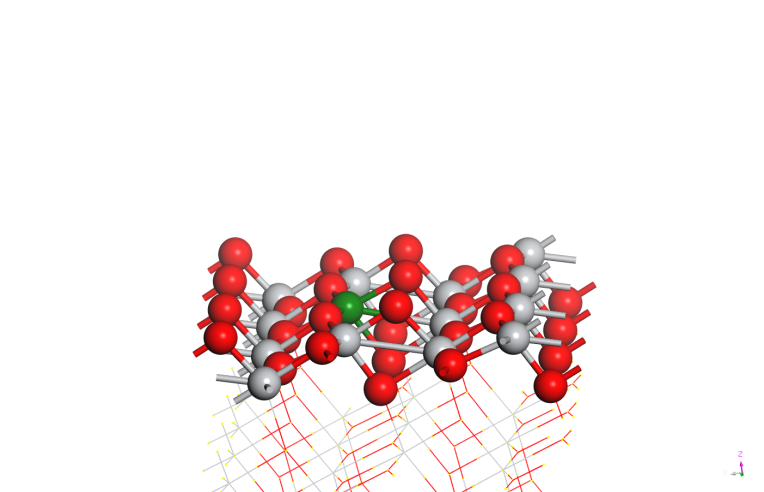

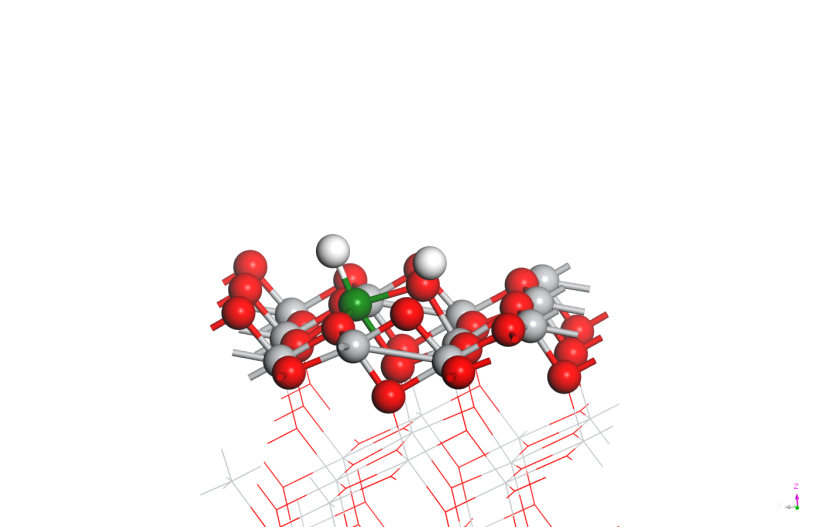

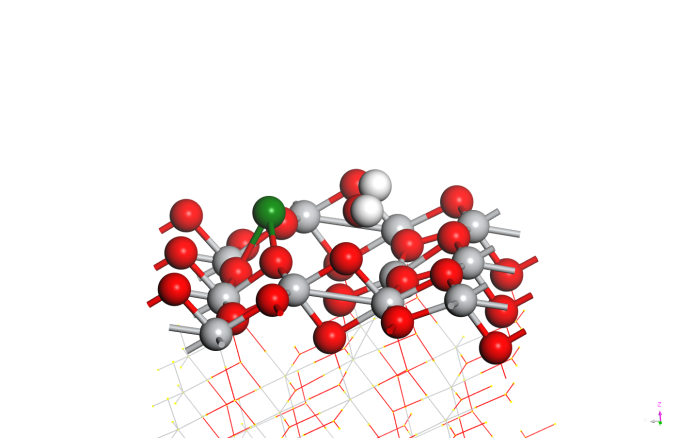

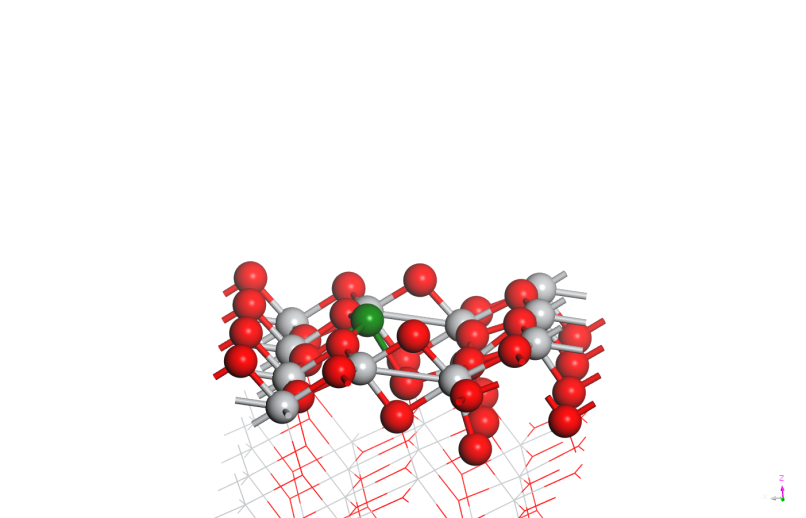

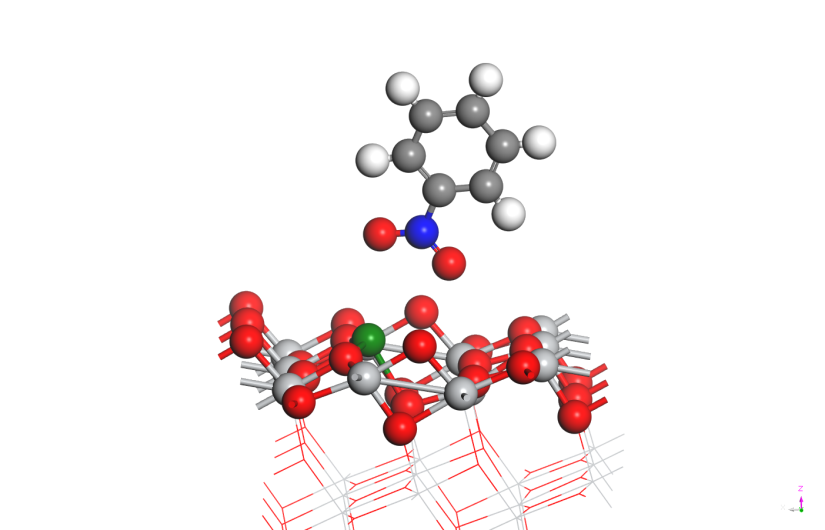

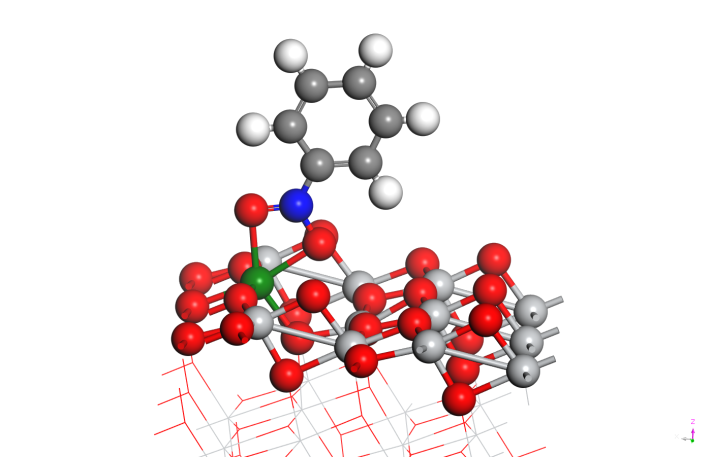

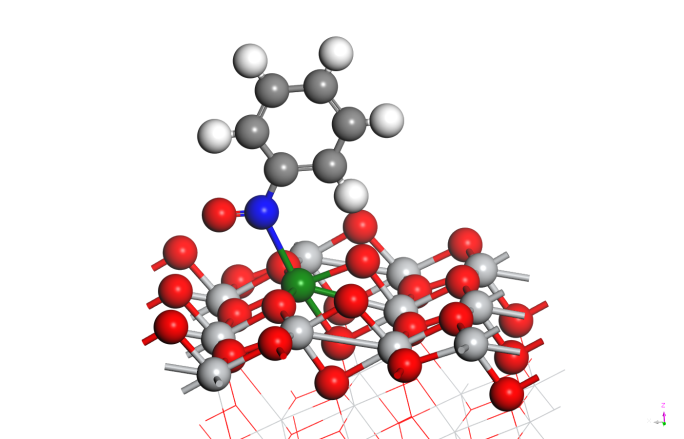

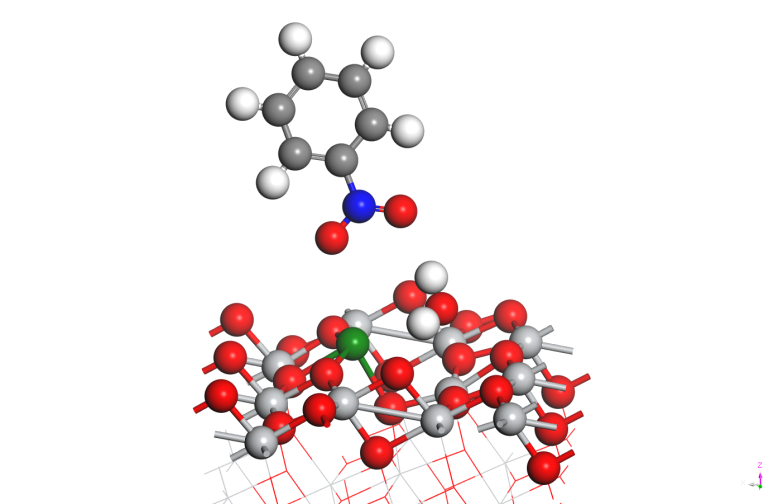


**C**

**B**

**A**

**F**

**E**

**D**


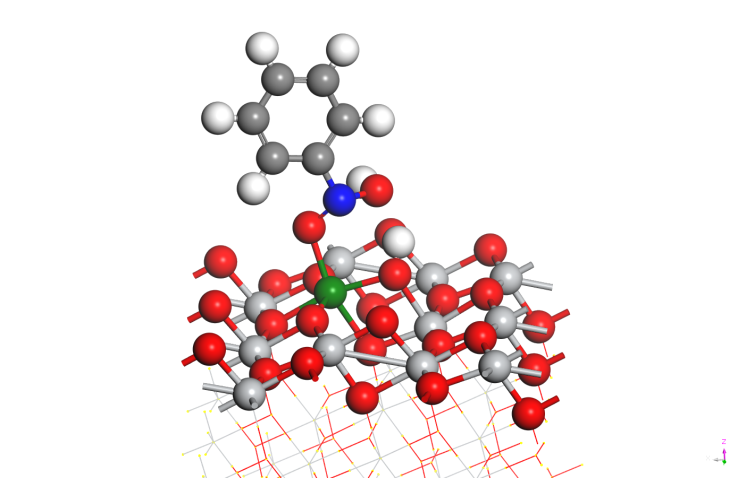


**I**

**H**

**G**

**Supplementary Figure 27.** Optimized structures of reactant and key intermediates in catalytic deoxygenation on the Sn_1_/TiO_2_(101) surface.

**Note:** Bader charge analysis showed that the charge on the Sn in **C** is +1.43 a.u., significantly lower than that in **A** (+2.34 a.u.), meaning that Sn has been reduced. Although these two steps were predicted to be exothermic, the corresponding barriers of **TS_A-B_** and **TS_B-C_** were as high as 0.77 eV and 1.20 eV (Figure 1), respectively, indicating that the formation of a surface oxygen vacancy still required a relatively high temperature. We stress that H_2_ could be easily dissociated on the noble metal surfaces (except Au) or at the noble metal/oxide interfaces (e.g., Au/TiO_2_), which facilitates the formation of the oxygen atom vacancies.


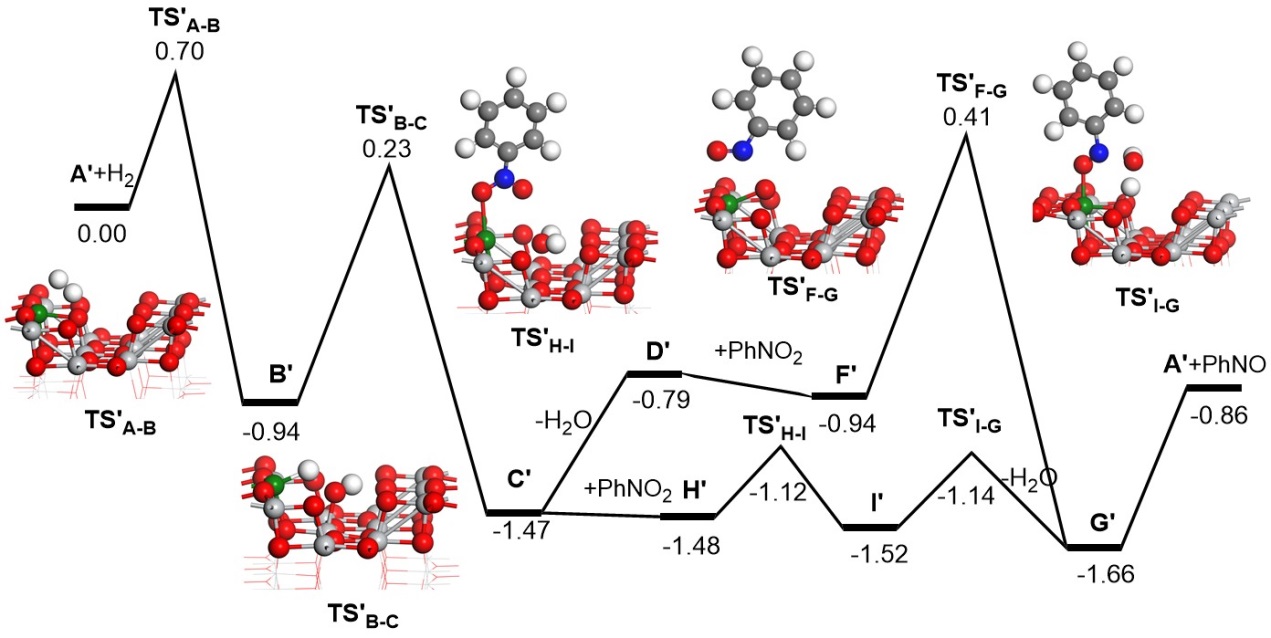


**Supplementary Figure 28.** Energy profile of catalytic deoxygenation of nitrobenzene over Sn_1_/TiO_2_(100) surface.

**Note:** We also investigated the deoxygenation of nitrobenzene on the Sn_1_/TiO_2_(100) surface. We found that the water-assisted deoxygenation mechanism was favored over the direct deoxygenation, in good agreement with the results characterizing reaction on the Sn_1_/TiO_2_(101) surface.


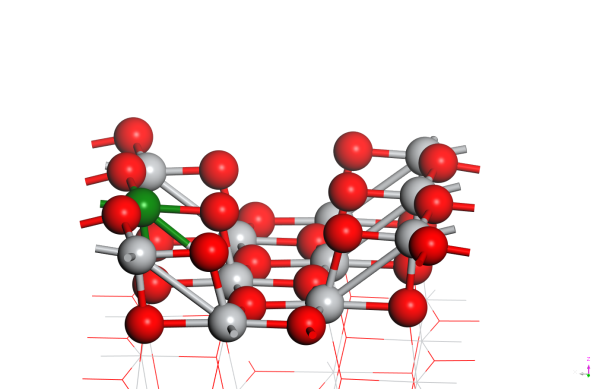

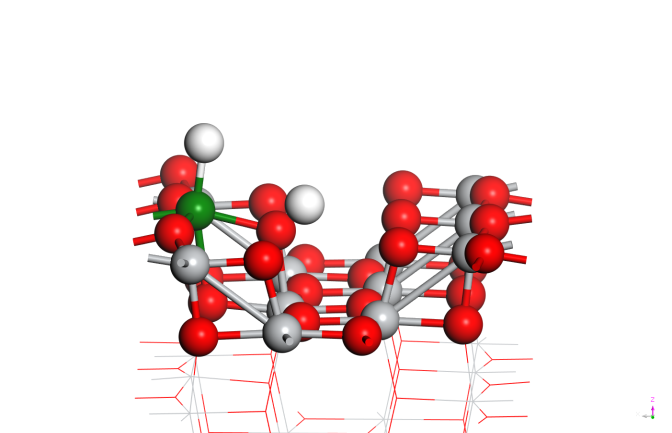

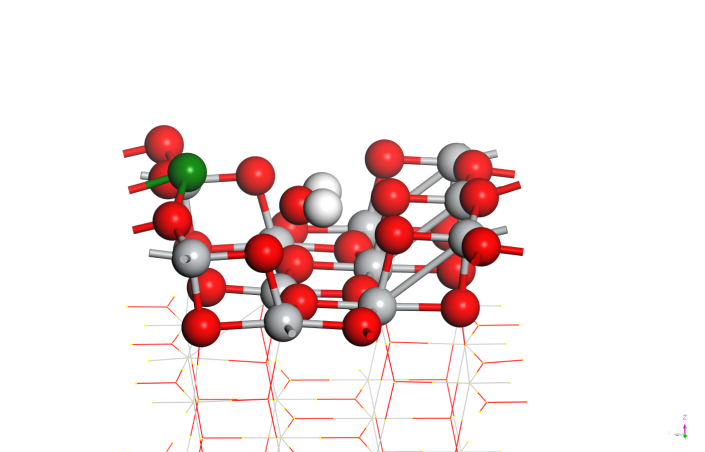

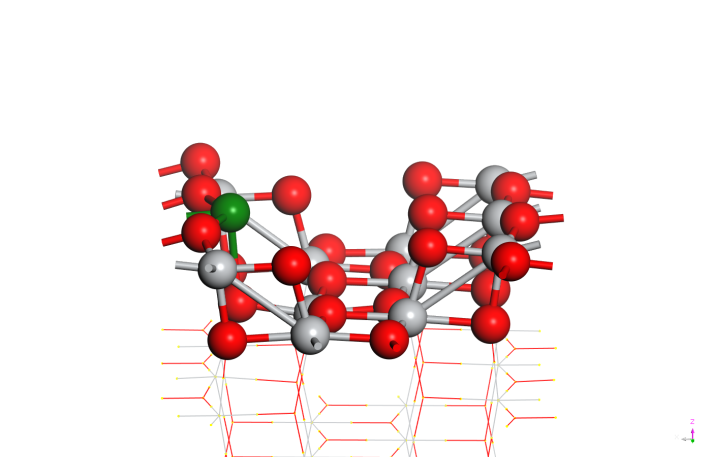

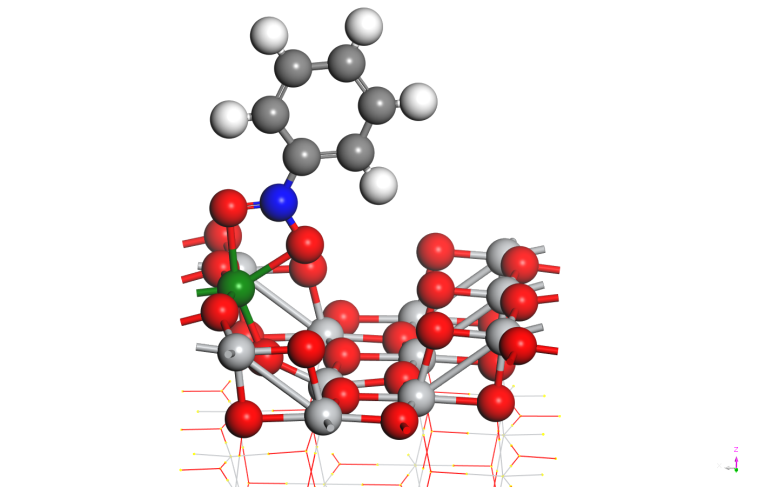

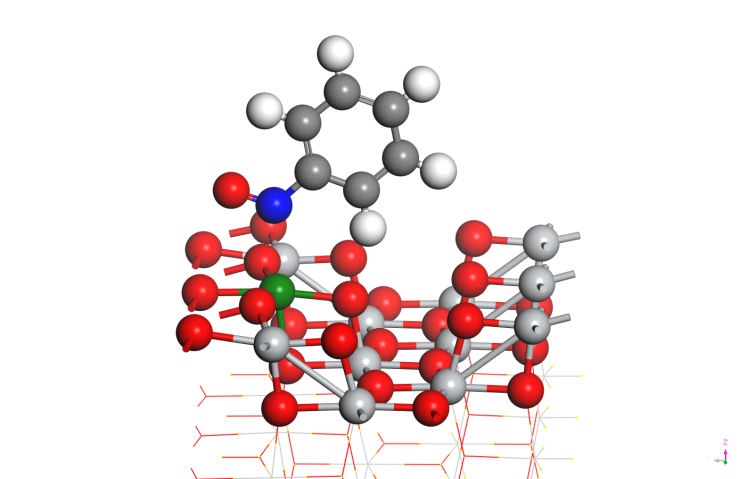


**C′**

**B′**

**A′**

**G′**

**F′**

**D′**


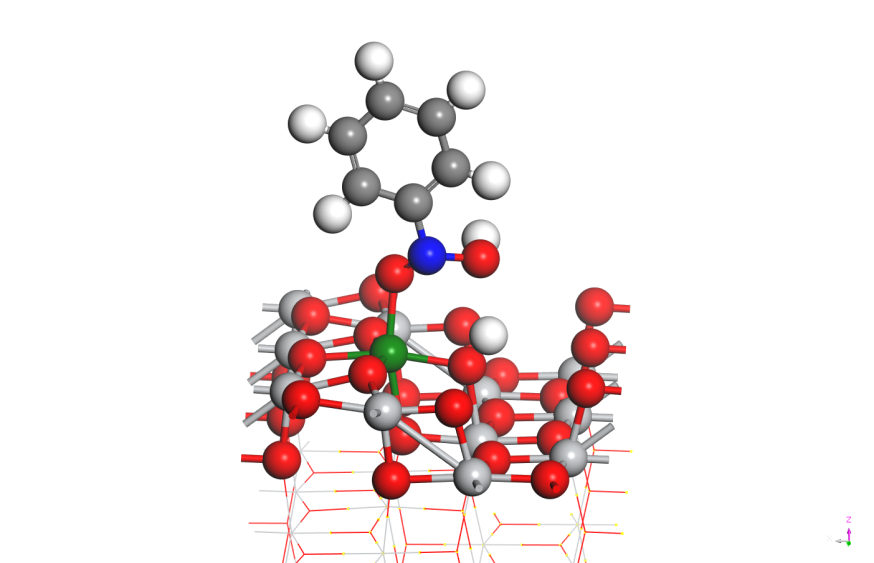

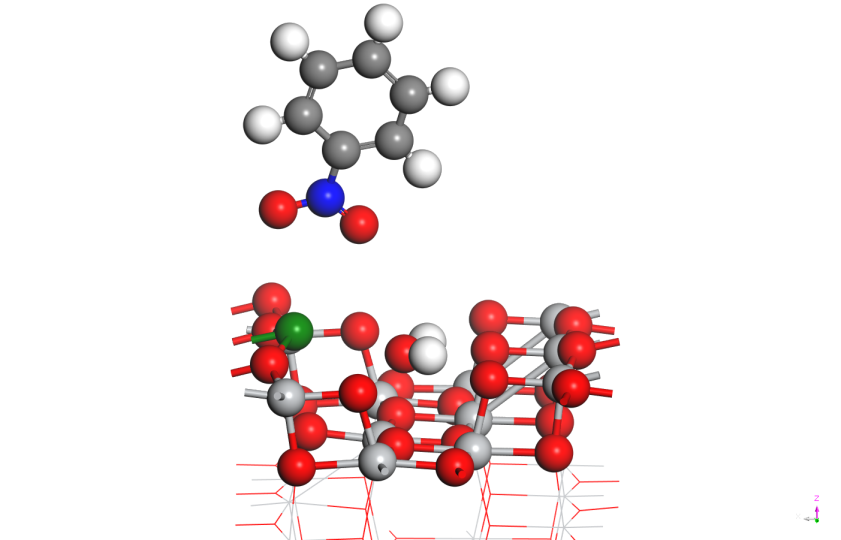


**H′**

**I′**

**Supplementary Figure 29.** Optimized structures of reactant and key intermediates in catalytic deoxygenation on the Sn_1_/TiO_2_(100) surface.

**Supplementary Table 2.** Metal loadings of the various catalyst samples.

cat, sub, solvent. 100 ^o^C, H_2_

| Sample | Molar ratio of Ti/Sn | Au loading (wt %) |
| --- | --- | --- |
| Au/TiO_2_ | --*^a^* | 0.8 |
| Au/Sn-TiO_2_-123 | 123 | 0.7 |
| Au/Sn-TiO_2_-20 | 20 | 0.7 |
| Au/Sn-SiO_2_-129 | 129*^b^* | 1.0 |
| Pt/TiO_2_ | --*^a^* | 0.4*^c^* |
| Pt/Sn-TiO_2_-123 | 123 | 0.4*^c^* |
| Ru/TiO_2_ | --*^a^* | 1.0*^d^* |
| Ru/Sn-TiO_2_-123 | 123 | 1.0*^d^* |
| Ni/TiO_2_ | --*^a^* | 2.8*^e^* |
| Ni/Sn-TiO_2_-123 | 123 | 2.9*^e^* |
| Au/Sn-TiO_2_-123*^f^* | 128 | 0.7 |

*^a^* Without tin; *^b^*molar ratio of Si/Sn; *^c^*platinum loading;

*^d^*ruthenium loading; *^e^*nickel loading;

*^f^*used catalyst after the seventh run.

**Supplementary Table 3.** EXAFS parameters characterizing tin foil and SnO_2_.

| Sample | Shell | N | R  (Å) |
| --- | --- | --- | --- |
| Tin foil*^a^* | Sn-Sn | 6 | 2.295 |
| SnO_2_*^b^* | Sn-O | 6 | 2.054 |
|  | Sn-Sn | 2 | 3.186 |

*^a^*The data are from Ref. 17; *^b^*the data are from Ref. 18.

*N*, coordination number; *R*, distance between absorber and backscatterer atoms

**Supplementary Table 4.** Catalytic data characterizing various gold catalysts in the hydrogenation of nitroarenes.*^a^*

| Entry | Catalyst | *T* (K) | Time (h) | Conversion (%) |  | Selectivity*^b^* (%) | N balance closure*^c^* (%) |
| --- | --- | --- | --- | --- | --- | --- | --- |
| 2-Chloro-4-nitrophenol reactant | | | | | | | |
| 1 | Au/TiO_2_ | 373 | 1.5 | 48.7 |  | 91.6 | 99.2 |
| 2 | Au/Sn-TiO_2_-123 | 373 | 1.5 | 94.6 |  | 99.1 | 99.0 |
| 3 | Au/Sn-TiO_2_-20 | 373 | 1.5 | 11.7 |  | 89.0 | >99.5 |
| 4 | Au/SnO_2_ | 373 | 1.5 | 6.3 |  | 80.1 | >99.5 |
| 4-Nitrobenzaldehyde reactant | | | | | | | |
| 5 | Au/TiO_2_ | 353 | 1.5 | 77.0 |  | 92.8 | 92.1*^d^* |
| 6 | Au/Sn-TiO_2_-123 | 353 | 1.5 | 99.0 |  | 93.1 | 92.4*^d^* |
| 4-Nitrochlorobenzene reactant | | | | | | | |
| 7 | Au/TiO_2_ | 363 | 2.0 | 44.3 |  | 99.1 | >99.5 |
| 8 | Au/Sn-TiO_2_-123 | 363 | 2.0 | 93.1 |  | 99.0 | >99.5 |
| 4-Nitrobenzamide reactant*^e^* | | | | | | | |
| 9 | Au/TiO_2_ | 363 | 2.5 | 71.8 |  | >99.5 | >99.5 |
| 10 | Au/Sn-TiO_2_-123 | 363 | 2.5 | >99.5 |  | >99.5 | >99.5 |
| 3-Nitrobenzonitrile reactant*^e^* | | | | | | | |
| 11 | Au/TiO_2_ | 373 | 4.0 | 66.6 |  | 93.0 | >99.5 |
| 12 | Au/Sn-TiO_2_-123 | 373 | 4.0 | >99.5 |  | 90.2 | >99.5 |

*^a^*Reaction conditions: 0.5 mmol of nitroarene, 40 mg of catalyst, 4 ml of toluene, 1.3 MPa partial pressure of H_2_; *^b^*selectivity to the functionalized anilines; *^c^*calculated from the number of N-containing molecules in the reactor before and after the reaction; *^d^*the lost N is attributed to the polymerization of 4-aminobenzaldehyde, which was not included in calculating the product selectivity;*^e^* 4 ml of THF was used as solvent with a 2.5 MPa partial pressure of H_2_.

**Supplementary Table 5.** Dispersion degree and TOFs of various catalysts.

| **Catalyst** | **Dispersion degree (%)** | **TOF (h^-1^)***^a^* | **TOF (h^-1^)***^b^* |
| --- | --- | --- | --- |
| Au/TiO_2_ *^c^* | 34 | 297 | 270 |
| Au/Sn-TiO_2_-123 *^c^* | 30 | 873 | 861 |
| Au/Sn-TiO_2_-20 *^c^* | 38 | 319 | 290 |
| Pt/TiO_2_ *^d^* | 46 | 1744 | 1120 |
| Pt/Sn-TiO_2_-123 *^d^* | 42 | 1802 | 1758 |

*^a^*TOFs calculated from the 3-nitrostyrene conversion on the basis of exposed Au or Pt atoms; *^b^*TOFs calculated from the 3-vinylaniline production on the basis of exposed Au or Pt atoms; *^c^*Reaction conditions: batch reactor, 10 mmol of 3-nitrostyrene, 8 mg of catalyst, 10 mL of toluene, 1.3 MPa of H_2_, reaction time of 15 min, 343 K; *^d^*Reaction conditions: batch reactor, 10 mmol of 3-nitrostyrene, 8 mg of catalyst, 10 mL of toluene, 0.2 MPa of H_2_, reaction time of 15 min, 311 K.

**Note:** In the hydrogenation of 3-nitrostyrene, the Au/Sn-TiO_2_-123 gives much higher TOFs calculated from both the 3-nitrostyrene conversion (873 h^-1^) and 3-vinylaniline (desired product) production rate (861 h^-1^) than Au/TiO_2_ (297 h^-1^ for 3-nitrostyrene conversion and 270 h^-1^ for 3-vinylaniline production) and Au/Sn-TiO_2_-20 (319 h^-1^ for 3-nitrostyrene conversion and 290 h^-1^ for 3-vinylaniline production), confirming the enhancement of activity of Au catalysts by single-site Sn.

Pt/Sn-TiO_2_-123 exhibits slightly higher TOF for the 3-nitrostyrene conversion than Pt/TiO_2_ (1802 VS 1744 h^-1^) and obviously enhanced TOF for 3-vinylaniline production (1758 VS 1120 h^-1^). These data confirm that the single-site Sn enhanced both the activity and selectivity of Pt catalysts.

**Supplementary Table 6.** Catalytic data characterizing various platinum, ruthenium, and nickel catalysts in the hydrogenation of 2-chloro-4-nitrophenol.*^a^*

| Entry | Catalyst | *T* (K) | Time (h) | Conversion (%) | Selectivity*^b^* (%) | N balance closure*^c^* (%) |
| --- | --- | --- | --- | --- | --- | --- |
| 2-Chloro-4-nitrophenol reactant | | | | | | |
| 1 | Pt/TiO_2_ | 333 | 3.0 | 81.9 | 56.0 | >99.5 |
| 2 | Pt/Sn-TiO_2_-123 | 333 | 3.0 | >99.5 | 99.0 | 99.1 |
| 3 | Ru/TiO_2_ | 363 | 6.0 | 90.0 | 70.3 | >99.5 |
| 4 | Ru/Sn-TiO_2_-123 | 363 | 6.0 | 93.1 | 95.2 | >99.5 |
| 5 | Ni/TiO_2_ | 393 | 6.0 | 26.0 | 35.1 | 99.0 |
| 6 | Ni/Sn-TiO_2_-123 | 393 | 6.0 | 59.4 | 96.5 | 99.0 |

*^a^*Reaction conditions: 0.5 mmol of nitroarene, 40 mg of catalyst, 4 ml of toluene, 0.7 MPa of H_2_ for platinum catalysts, 1.3 MPa partial pressure of H_2_ for ruthenium catalysts and 2.5 MPa partial pressure of H_2_ for nickel catalysts; *^b^*selectivity to the functionalized anilines; *^c^*calculated from the number of N-containing molecules in the reactor before and after the reaction.

**Supplementary Table 7.** TOFs in the hydrogenation of nitrobenzene and styrene at various ratios of reactant to catalyst (S/C) on the Pt/TiO_2_ and Pt/Sn-TiO_2_-123 catalysts.*^a^*

.

| Catalyst | Feed (S/C) | | TOF (mol_converted_ mol_Pt_^-1^ h^-1^) | |
| --- | --- | --- | --- | --- |
|  |  |  |  |
| Pt/TiO_2_ | 2500 | 0 | 1206 | - |
| Pt/TiO_2_ | 0 | 2500 | - | 1608 |
| Pt/TiO_2_ | 1250 | 1250 | 1010 | 905 |
| Pt/Sn-TiO_2_-123 | 2500 | 0 | 1917 | - |
| Pt/Sn-TiO_2_-123 | 0 | 2500 | - | 1262 |
| Pt/Sn-TiO_2_-123 | 1250 | 1250 | 2150 | 134 |

*^a^*Reaction conditions: 10 mmol of substrate, 20 mL of toluene, 318 K, and 0.2 MPa of H_2_. The TOFs were calculated on the basis of exposed Pt atoms on the catalysts.

**Note:** The table presents the turnover frequency values (TOFs) calculated from the initial reaction rates as the number of molecules transformed per hour per Pt atom at various ratios of reactant (substrate) to catalyst (S/C) in the competitive hydrogenation of -NO_2_ and -C=C groups by employing nitrobenzene and styrene as model reactant molecules for reaction on the TiO_2_ supported Pt nanoparticle catalysts with and without Sn promotion. Notably, both Pt/TiO_2_ and Pt/Sn-TiO_2_-123 catalysts are active for the hydrogenation of nitrobenzene and styrene. However, when a mixture of nitrobenzene and styrene was used, the hydrogenation of styrene was strongly inhibited (TOF of 1262 with styrene reactant VS 134 with mixed reactants) in the presence of nitrobenzene over the Pt/Sn-TiO_2_-123 catalyst. In contrast, the hydrogenation of styrene was slightly influenced (TOF of 1608 with styrene reactant vs. 905 with mixed reactants) in the presence of nitrobenzene on the Pt/TiO_2_ catalyst. These data confirm that the hydrogenation of -C=C group was strongly hindered by the presence of -NO_2_ groups, and this result is in good agreement with the observed high selectivity for vinyl aniline in hydrogenation of nitrostyrene.

**Supplementary Table 8.** Calculated adsorption energies of N-containing compounds and H_2_ on the low Miller-index surfaces of Au and Pt.

| Adsorption energies (eV) | | | | | | | |
| --- | --- | --- | --- | --- | --- | --- | --- |
| Compound | gold | | |  | Pt | | |
|  | (111) | (100) | (110) |  | (111) | (100) | (110) |
| Nitrobenzene | -0.04 | -0.08 | -0.27 |  | -1.05 | -1.97 | -2.10 |
| Nitrosobenzene | -0.19 | -0.38 | -0.61 |  | -1.31 | -1.79 | -1.59 |
| Hydroxyl aniline | -0.19 | -0.33 | -0.43 |  | -0.90 | -0.99 | -1.19 |
| Aniline | -0.19 | -0.12 | -0.36 |  | -0.82 | -0.86 | -1.05 |
| H_2_^a^ | 0.20 | 0.10 | -0.08 |  | -0.99 | -1.33 | -1.20 |

*^a^*Dissociative adsorption

**Supplementary Table 9.** Calculated hydrodeoxygenation energy (Δ*E*_HDO_) for TiO_2_ and Sn_1_/TiO_2_ surfaces.

| Catalyst | Surface | Hydrodeoxygenation energy (eV) |
| --- | --- | --- |
| TiO_2_ | (101) | 1.46 |
|  | (110) | 0.83 |
|  | (011) | 0.20 |
| Sn_1_/TiO_2_ | (101) | -0.88 |
|  | (110) | -0.79 |
|  | (011) | -1.39 |

**Note:** Consider that both TiO_2_ and SnO_2_ are reducible oxides which would generate oxygen vacancies under the hydrogenation conditions. From the viewpoint of thermodynamics, the resulting oxygen vacancies could abstract an O atom from nitrobenzene to yield nitrosobenzene if they had suitable binding strength to O atoms. Here, we defined the hydrodeoxygenation energy (Δ*E*_HDO_) as the energy cost (or gain) of removing an oxygen atom by H_2_ from substrate or oxide surfaces. According to our calculations, the Δ*E*_HDO_ for nitrobenzene was -0.86 eV. Thus, an appropriate Δ*E*_HDO_ for the oxide supports should be in the range of -0.86 eV to 0.00 eV.

For the unmodified TiO_2_, the Δ*E*_HDO_ for the two-coordinated bridge oxygen (O_br_) on (101), (110), and (001) facets were 1.46, 0.83, and 0.20 eV, respectively (Supplementary Table 9). However, when a surface Ti_5c_^4+^ was substituted by a Sn_5c_^4+^ (Sn_1_/TiO_2_), the ΔE_HDO_ for the nearby O_br_ decreased significantly, giving -0.88, -0.79, and -1.39 eV for Sn_1_/TiO_2_(101), Sn_1_/TiO_2_(100), and Sn_1_/TiO_2_(001), respectively. Thus, Sn_1_/TiO_2_(101) and Sn_1_/TiO_2_(100) were expected to be catalysts for the hydrodeoxygenation of nitrobenzene.

**Supplementary References:**

1 Newville, M., Ravel, B., Haskel, D., Rehr, J. J., Stern, E. A., Yacoby, Y. Analysis of multiple-scattering XAFS data using theoretical standards. *Physica B.* **208**, 154-156 (1995).

2 Newville, M. EXAFS analysis using FEFF and FEFFIT. *J Synchrotron Radiat.* **8**, 96-100 (2001).

3 Vaarkamp, M., Linders, J. C., Koningsberger, D. C. A new method for parameterization of phase shift and backscattering amplitude. *Physica B.* **208**, 159-160 (1995).

4 Uzun, A., Bhirud, V. A., Kletnieks, P. W., Haw, J. F., Gates, B. C. A site-isolated iridium diethylene complex supported on highly dealuminated Y zeolite: synthesis and characterization. *J. Phys. Chem. C* **111**, 15064-15073 (2007).

5 Koningsberger, D. C., Mojet, B. L., Van Dorssen, G. E., Ramaker, D. E. XAFS spectroscopy; fundamental principles and data analysis. *Top. Catal.* **10**, 143-155 (2000).

6 Kresse, G., Furthmüller, J. Efficient iterative schemes for ab initio total-energy calculations using a plane-wave basis set. *Phys. Rev. B* **54**, 11169 (1996).

7 Kresse, G., Furthmüller, J. Efficiency of ab-initio total energy calculations for metals and semiconductors using a plane-wave basis set. *Comput. Mater. Sci.* **6**, 15 (1996).

8 Kresse, G., Hafner, J. Ab initio molecular dynamics for open-shell transition metals. *Phys. Rev. B* **48**, 13115 (1993).

9 Kresse, G., Hafner, J. Ab initio molecular-dynamics simulation of the liquid-metal–amorphous-semiconductor transition in germanium. *Phys. Rev. B* **49**, 14251 (1994).

10 Perdew, J. P., Burke, K., Ernzerhof, M. Generalized gradient approximation made simple. *Phys. Rev. Lett.* **77**, 3865 (1996).

11 Blöchl, P. E. Projector augmented-wave method. *Phys. Rev. B* **50**, 17953-17979 (1994).

12 Kresse, G. & Joubert, D., D. From ultrasoft pseudopotentials to the projector augmented-wave method. *Phys. Rev. B* **59**, 1758-1775 (1999).

13 Naidu, H. P., Virkar, A. V. Low-Temperature TiO_2_-SnO_2_ Phase Diagram Using the Molten-Salt Method. *J. Am. Ceram. Soc.* **81**, 2176–2180 (1998).

14 Bader, R. F. W. Atoms in molecules: A quantum theory. New York: Oxford University Press. (1990)

15 Izumi, Y., Nagamori, H.; Kiyotaki, F.; Masih, D.; Minato, T.; Roisin, E.; Candy, J.-P.; Tanida, H.; Uruga, T. X-ray Absorption Fine Structure Combined with X-ray Fluorescence Spectrometry: Improvement of Spectral Resolution at the Absorption Edges of 9—29 keV. *Anal. Chem.* **77**, 6969-6975 (2005).

16 Masai, H., Miyata, H., Yamada, Y., Okumura, S., Yanagida, T., Kanemitsu, Y. Tin-Doped Inorganic Amorphous Films for Use as Transparent Monolithic Phosphors. *Sci. Rep.* **5**, 11224 (2015).

17 Wyckoff, R. W. G., *Crystal Structures* (Wiley-VCH, New York, 1963).

18 Bolzan, A. A., Gong, C., Kennedy, B. Structural studies of rutile-type metal dioxides. *Acta Crystallogr. B* **53**, 373-380 (1997).
